# Visible-Light-Mediated Ring-Opening Geminal Dibromination of Alkenes via Alkoxy Radicals Enabled by Electron Donor–Acceptor Complex

**DOI:** 10.3390/molecules29143281

**Published:** 2024-07-11

**Authors:** Rong Wei, Yuan Wang, Juantao Zhang, Chunsheng Wu, Zhenhua Zhang, Duo Zhang

**Affiliations:** 1 State Key Laboratory for Performance and Structure Safety of Petroleum Tubular Goods and Equipment Materials, Tubular Goods Research Institute of CNPC, Xi’an 710077, China; weir@cnpc.com.cn (R.W.); 202232655@stumail.nwu.edu.cn (Z.Z.); 2 National Engineering Laboratory of Low Permeability Oil-Gas Field Exploration and Development, Changqing Oilfield, Xi’an 710018, China; wucs01_cq@petrochina.com.cn; 3 Medicine Center, Guangxi University of Science and Technology, Liuzhou 545006, China; duo.zhang@gxust.edu.cn

**Keywords:** geminal dibromination, electron donor–acceptor complex, alkoxy radical, photochemistry, hypervalent iodine compounds

## Abstract

An electron donor–acceptor complex was utilized to generate alkoxy radicals from alcohols under mild conditions using visible light. This approach was combined with a hydroxybromination process to achieve the deconstructive functionalization of alkenes, leading to the production of geminal dibromides. Mechanistic investigations indicated the intermediacy of hypervalent iodine (III) compounds.

## 1. Introduction

In organic photochemistry, electron donor–acceptor (EDA) complexes play an important role in facilitating photochemical reactions [1,2]. When an electron donor and acceptor form a complex, they can absorb visible light and undergo single-electron transfer (SET). Despite being unable to absorb visible light individually, EDA complexes create radical intermediates to participate in subsequent reactions. Electron donor–acceptor (EDA) complexes in photochemistry offer a cost-effective alternative to obtain target chemical structures without relying on expensive photocatalysts [3,4,5,6]. Despite significant progress made in recent years regarding the potential of EDA complexes in synthetic methods [7,8,9,10,11,12,13,14,15,16,17], further exploration of the diversity in EDA complexes is needed to fully realize the potential of this approach for manipulating radical chemistry. Expanding the range of EDA complexes could open up broader prospects in this field.

**Figure 1:**
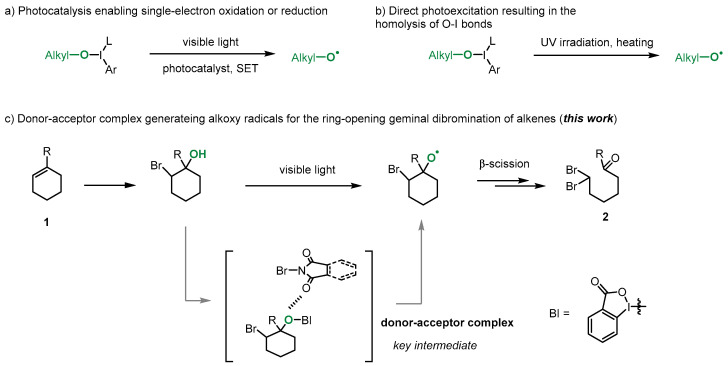
Alkoxyl radical generation based on O-I bond.

Alkoxy radicals are highly reactive open-shell species that are utilized as crucial intermediates in various synthetic strategies [18,19,20,21]. However, generating alkoxy radicals from alcohols under mild conditions remains challenging and continues to attract the attention of researchers [22,23,24,25,26,27,28]. Recent developments in photochemistry have made it possible to convert free alcohols to alkoxy radicals under mild conditions [29,30,31,32,33,34,35,36,37,38,39]. Previous reports have shown that hypervalent iodine (III) compounds can facilitate the in situ formation of O-I (III) intermediates that can be easily cleaved to produce alkoxy radicals via single-electron oxidation or reduction by photocatalysts or direct photoexcitation, resulting in the homolysis of O-I bonds [40,41,42,43] (Figure 1a,b). In this study, we developed a method for the visible-light-promoted generation of alkoxy radicals using a donor–acceptor complex, which was used to achieve the ring-opening geminal debromination of alkenes. Alkene **1** first reacted with NBS in the presence of H_2_O, forming a hydroxybromation adduct in situ, which was converted to an alkoxy radical via the EDA complex in the reaction mixture. The subsequent β-scission process could provide a ring-opening process, resulting in the final product **2** [44] (Figure 1c).

## 2. Results and Discussion

**Figure 2:**
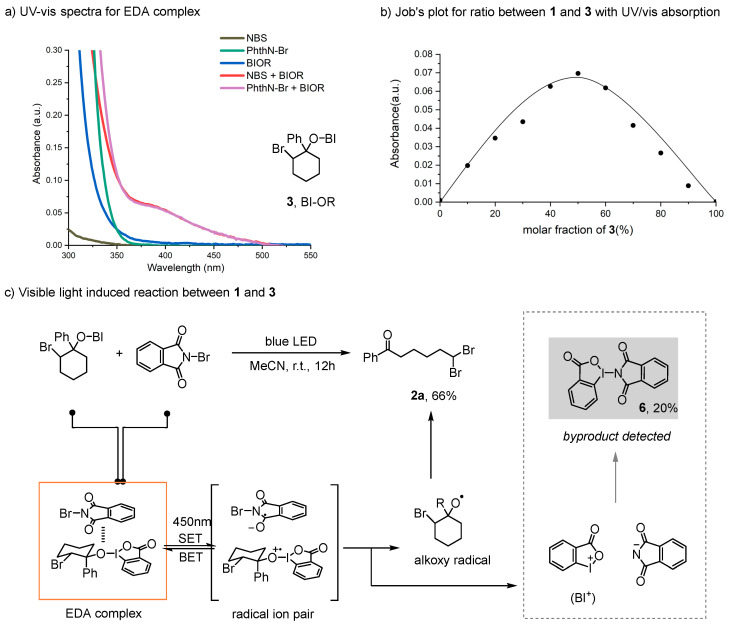
EDA complex studies and reaction design.

Our investigation began with the use of iodine (III) benziodoxole precursor **3** with commercially available N-bromoimides (N-bromosuccinimide **4** and N-bromophthalimide **5**). Upon mixing **3** with N-bromosuccinimide or N-bromophthalimide, the UV/vis absorption spectrum of the mixture showed significant red-shifts (Figure 2a). A Job plot was also performed to confirm the formation of the donor–acceptor complex in the mixture (Figure 2b) [9]. Subsequently, when iodine (III) benziodoxole **3** and **5** were exposed to blue light, the ring-opening product **2a** was obtained in a 66% yield (Figure 2c). In this process, the EDA complex was photoactivated by blue light, and an intracomplex single-electron transfer (SET) afforded the radical ion pair. Then, by facilitating the irreversible cleavage of the O-I bond, the radical ion pair released the alkoxy radical, avoiding the back electron transfer (BET) process. Additionally, the by-product 6 was detected in the reaction mixture and could be isolated in a 20% yield, likely produced from the BI+ species during the reaction process. Previous reports have shown that BI-OR species can react with photocatalysts via a single-electron transfer (SET) process, generating the corresponding oxy radical and BI+ in reactions. Specifically, BI-OR species have been reported to undergo single-electron oxidation in reactions [32,33,35]. These observations, along with previous reports, suggest that N-bromophthalimide may act as the electron acceptor in this case, with compound 3 serving as the electron donor. This is consistent with previous studies on similar iodine (III) species [32,33,34,35,45,46,47,48].

We then applied this method of alkoxy radical generation to the cascade ring-opening geminal dibromination reaction. Phenyl-1-cyclohexene **1a** was selected as a representative model substrate and reacted as hypothesized. In order to raise the yield of product **2a**, various modifications were tested (Table 1). Using an excess of the hypervalent iodine reagent to generate the BI-OR intermediate showed good results. When 1.5 equiv of BIOAc was added to the reaction mixture, **2a** was obtained in a 39% yield. The usage of 2 equiv of BIOAc led to a good yield of 54%. Using NBS as a cheaper alternative in the reaction process, the corresponding product was obtained in a similar yield of 52%. Without a cooling fan, the reaction temperature was raised to 50 °C, which led to a higher yield of 76%. Changing the solvent from MeCN to DCM, PhCl, Acetone, THF, or EtOAc had no positive effect on the yield of the target product. Finally, prolonging the reaction time to 36 h gave a yield of 83%, and the control experiment confirmed that light irradiation was essential for this process.

**Figure 3:**
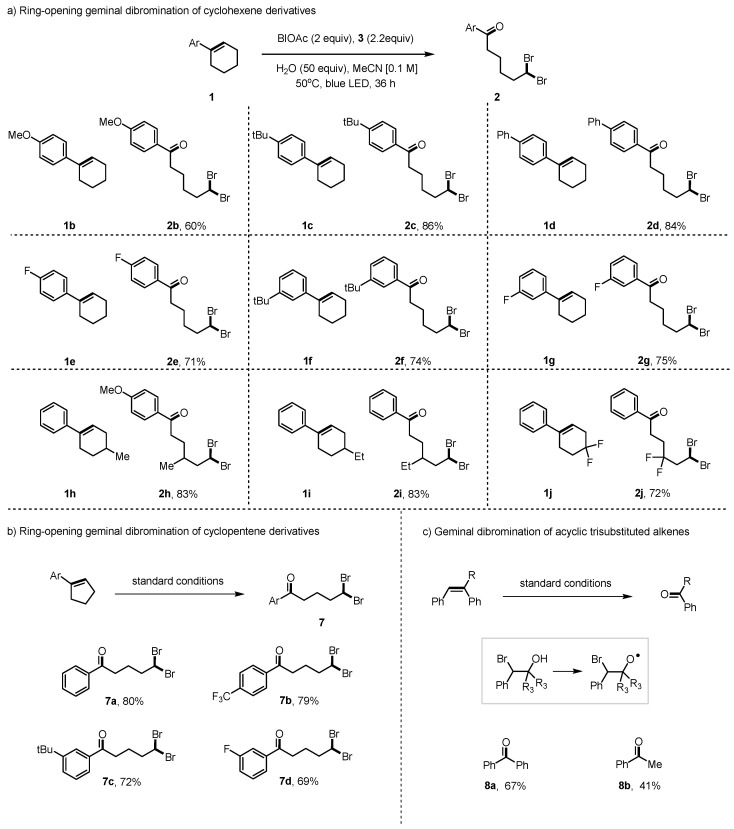
Geminal dibromination of alkenes.

With the optimized reaction conditions in hand, the substrate scope of this ring-opening geminal dibromination was investigated. In this context, a variety of cyclohexene derivatives were applied. As shown in Figure 3, the aryl group functionality was examined, and all reactions proceeded well, resulting in the corresponding product **2a**–**h** in moderate-to-good yields. Products **2b**–**f** contained aryl rings with varying electronic groups in the para-positions that were tolerant in the reaction process and gave a moderate-to-good yield of 60–84%. Products **2c** and **2d,** containing electron-donating groups, could be isolated in higher yields as compared to **2e,** which contained electron-withdrawing groups. As product **2b** contained a methoxy group within the structure, an aryl group bromination by-product was also detected [49,50,51], resulting in a lower yield in comparison to other substrates. Cyclohexenes **2** with substituents on the meta-positions of the phenyl group were also examined, affording target products in good yields. Then, various substituents on the cyclohexene rings were also studied with no drastic differences in the yield for products **2h**–**j**. The cyclohexene rings were modified with methyl, ethyl, and difluoro groups (**2h**–**j**), and the results indicated that neither electronic nor steric effects had a significant influence on the procedure.

Apart from using cyclohexene derivatives, we also studied the productivity of this method with cyclopentene derivatives containing various functional groups on the aryl rings, as shown in Figure 3b,c. The corresponding products **7a**–**d** were obtained in 69–80% yields. Moreover, this method was further examined with acyclic trisubstituted alkenes, which were transformed to the ketone 8a and 8b successfully by β-scission [29,52].

**Figure 4:**
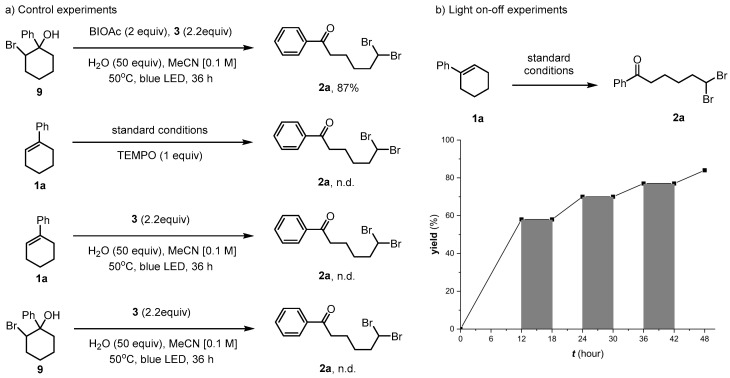
Control experiments and light on–off study.

To gain mechanistic insight, we used bromohydrin **9** under standard conditions, which led to the formation of **2a** [53]. No target product was obtained in the presence of TEMPO, implying that a radical step might have been involved in the reaction mechanism. Neither **1a** nor **9** could be converted to **2a** in the absence of BI-OAc. The results of the on–off experiment are shown in Figure 4b. When the light was switched off, the formation of **2a** immediately stopped. Conversely, the conversion from **1a** to **2a** resumed upon turning on the light, which excluded the possibility of a radical chain process in this reaction.

## 3. Materials and Methods

### 3.1. General Information

Unless otherwise noted, all chemicals were purchased from commercial suppliers (Sigma Aldrich (St. Louis, MO, USA), TCI (Toyko, Japan), Oakwood (West Columbia, SC, USA)) and used without further purification. When required, solvents were dried according to general purification methods. Two Kessil PR160 blue LED lamps (40 W), purchased from Kessil (Richmond, VA, USA), were used as the light source. The Emission profile of the Kessil PR160 blue LED lamps used to irradiate the reaction mixture is shown in Figure S2. The product mixtures were analyzed by thin-layer chromatography using TLC silica gel plates (Merck-Schuchardt, Hohenbrunn, Germany) with a fluorescent indicator (λ = 254 nm). The purification of the products was performed by flash column chromatography using silica gel 60 (63–200 µm) from SANPONT (Shanghai, China). NMR spectra were recorded on a Bruker AV-III400 (400 MHZ) or AMX500 (500 MHz) spectrometer (Karlsruhe, Germany) in deuterated solvents. Chemical shifts (δ) are reported in parts per million (ppm), and spin–spin coupling constants (J) are given in Hz, while multiplicities are given the standard abbreviations, s (singlet), d (doublet), t (triplet), q (quartet), br (broad), and m (multiplet). High-resolution mass spectra (HRMS) were recorded on a Finnigan/MAT 95XL-T spectrometer (San Jose, CA, USA). The diastereomeric ratio (dr) was determined by 1H NMR of the crude product mixture. Absorption spectra were recorded in 1 cm path quartz cuvettes using an Edinburgh FS-5 spectrofluorometer (Livingston, UK). Continuous wave X-band ESR spectra were obtained with a JEOL (FA200) spectrometer (Toyko, Japan).

### 3.2. General Procedure for the Photocatalytic Oxidation Reaction

Alkene (0.2 mmol, 1.0 equiv), BIOAc (0.4 mmol, 2.0 equiv), N-bromophthalimide (0.44 mmol, 2.2 equiv), H_2_O (10 mmol, 50.0 equiv), and MeCN (2.0 mL) were added to a schlenk tube (10 mL) equipped with a magnetic stirring bar. Then, the reaction mixture underwent freeze–pump–thaw procedures three times and were backfilled with argon. The resulting solution was magnetically stirred at 50 °C and irradiated using two 40 W Kessil PL160L lamps (456 nm, purchased from Kessil, Richmond, VA, USA). After 36 h, the reaction solution was concentrated, and the product was purified by column chromatography (SiO_2_). (Note: Reactions were irradiated using two 40 W Kessil PR160 lamps (456 nm, purchased from Kessil, Richmond, VA, USA) while stirring on stir plates. The reactions were placed approximately 5 cm away from the LEDs, and an external cooling fan was used to ensure that temperatures ranged from 48 to 52 °C on average.)

*6,6-dibromo-1-phenylhexan-1-one* (**2a**). Yellow viscous oil (55.5 mg, 83% yield). ^1^H NMR (400 MHz, Chloroform-d) δ 8.03–7.84 (m, 2H), 7.63–7.53 (m, 1H), 7.48–7.40 (m, 2H), 5.73 (t, *J* = 6.2 Hz, 1H), 3.02 (t, *J* = 7.2 Hz, 2H), 2.57–2.38 (m, 2H), 1.88–1.72 (m, 2H), 1.71–1.64 (m, 2H). ^13^C NMR (126 MHz, Chloroform-d) δ 199.7, 136.9, 133.1, 128.7, 128.1, 45.8, 45.3, 38.2, 27.9, 22.8. HRMS *m*/*z*: calcd for C_12_H_14_Br_2_ONa (M + Na^+)^ = 354.9304, found 354.9305.

*6,6-dibromo-1-(4-methoxyphenyl)hexan-1-one* (**2b**). Yellow viscous oil (43.7 mg, 60% yield). ^1^H NMR (400 MHz, Chloroform-d) δ 7.94 (d, *J* = 8.9 Hz, 2H), 6.94 (d, *J* = 8.9 Hz, 2H), 5.72 (t, *J* = 6.2 Hz, 1H), 3.87 (s, 3H), 2.96 (t, *J* = 7.2 Hz, 2H), 2.56–2.31 (m, 2H), 1.90–1.71 (m, 2H), 1.71–1.58 (m, 2H). ^13^C NMR (126 MHz, CDCl3) δ 198.4, 163.6, 130.4, 130.2, 113.9, 55.6, 45.9, 45.4, 37.9, 28.0, 23.1. HRMS *m*/*z*: calcd for C_13_H_16_Br_2_O_2_Na (M + Na^+^) = 384.9409, found 384.9409.

*6,6-dibromo-1-(4-(tert-butyl)phenyl)hexan-1-one* (**2c**). Yellow viscous oil (67.1 mg, 86% yield). ^1^H NMR (500 MHz, Chloroform-d) δ 7.99–7.73 (m, 2H), 7.62–7.42 (m, 2H), 5.73 (t, *J* = 6.2 Hz, 1H), 2.99 (t, *J* = 7.2 Hz, 2H), 2.58–2.29 (m, 2H), 1.92–1.68 (m, 2H), 1.69–1.56 (m, 2H), 1.34 (s, 9H). ^13^C NMR (126 MHz, CDCl3) δ 199.3, 156.8, 134.4, 128.0, 125.6, 125.5, 45.7, 45.2, 38.0, 35.1, 31.1, 27.8, 22.9. HRMS *m*/*z*: calcd for C_16_H_12_Br_2_ONa (M + Na^+^) = 410.9930, found 410.9933.

*1-([1,1′-biphenyl]-4-yl)-6,6-dibromohexan-1-one* (**2d**). Yellow viscous oil (68.9 mg, 84% yield). ^1^H NMR (400 MHz, Chloroform-d) δ 8.07–8.00 (m, 2H), 7.71–7.56 (m, 4H), 7.51–7.45 (m, 2H), 7.42–7.36 (m, 1H), 5.74 (t, *J* = 6.2 Hz, 1H), 3.05 (t, *J* = 7.2 Hz, 2H), 2.53–2.42 (m, 2H), 1.87–1.79 (m, 2H), 1.73–1.61 (m, 2H). ^13^C NMR (126 MHz, CDCl3) δ 199.2, 145.8, 139.9, 135.6, 132.1, 129.0, 128.6, 128.3, 127.3, 45.7, 45.2, 38.2, 27.8, 22.8. HRMS *m*/*z*: calcd for C_18_H_19_Br_2_O (M + H^+^) = 408.9797, found 408.9796.

*6,6-dibromo-1-(4-fluorophenyl)hexan-1-one* (**2e**). Yellow viscous oil (50.0 mg, 71% yield). ^1^H NMR (400 MHz, Chloroform-d) δ 8.05–7.93 (m, 2H), 7.18–7.08 (m, 2H), 5.73 (t, *J* = 6.1 Hz, 1H), 2.99 (t, *J* = 7.2 Hz, 2H), 2.50–2.36 (m, 2H), 1.87–1.71 (m, 2H), 1.70–1.62 (m, 2H). ^13^C NMR (126 MHz, CDCl3) δ 198.0, 166.8, 164.7, 133.3, 130.7, 130.6, 115.8, 115.6, 77.3, 77.0, 76.8, 45.6, 45.2, 38.0, 27.7, 22.7. HRMS *m*/*z*: calcd for C_12_H_14_Br_2_FO (M + H^+^) = 350.9390, found 350.9390.

*6,6-dibromo-1-(3-(tert-butyl)phenyl)hexan-1-one* (**2f**). Yellow viscous oil (57.7 mg, 74% yield). 1H NMR (500 MHz, Chloroform-d) δ 8.05–7.92 (m, 1H), 7.81–7.71 (m, 1H), 7.65–7.55 (m, 1H), 7.46–7.33 (m, 1H), 5.73 (t, *J* = 6.1 Hz, 1H), 3.02 (t, *J* = 7.2 Hz, 2H), 2.46 (ddd, *J* = 9.7, 7.0, 5.5 Hz, 2H), 1.90–1.76 (m, 2H), 1.72–1.61 (m, 2H), 1.36 (s, 9H). 13C NMR (126 MHz, CDCl3) δ 200.0, 151.8, 136.8, 130.2, 128.3, 125.4, 124.7, 45.7, 45.2, 38.2, 34.9, 31.3, 27.8, 22.8. HRMS *m*/*z*: calcd for C16H23Br2O (M + H^+^) = 389.0110, found 389.0115.

*6,6-dibromo-1-(3-fluorophenyl)hexan-1-one* (**2g**). Yellow viscous oil (52.8 mg, 75% yield). ^1^H NMR (500 MHz, Chloroform-d) δ 7.75–7.71 (m, 1H), 7.66–7.60 (m, 1H), 7.49–7.40 (m, 1H), 7.31–7.23 (m, 1H), 5.73 (t, *J* = 6.1 Hz, 1H), 3.00 (t, *J* = 7.2 Hz, 2H), 2.48–2.40 (m, 2H), 1.81 (dt, *J* = 15.1, 7.3 Hz, 2H), 1.70–1.60 (m, 2H). ^13^C NMR (126 MHz, CDCl3) δ 198.3, 163.9, 161.9, 139.0, 138.9, 130.3, 130.3, 123.7, 123.7, 120.2, 120.0, 114.9, 114.7, 45.6, 45.1, 38.3, 27.7, 22.6. HRMS *m*/*z*: calcd for C_12_H_14_Br_2_FO (M + H^+^) = 350.9390, found 350.9391.

*6,6-dibromo-1-(4-methoxyphenyl)-4-methylhexan-1-one* (**2h**). Yellow viscous oil (57.8 mg, 83% yield). ^1^H NMR (400 MHz, Chloroform-d) δ 8.04–7.90 (m, 2H), 7.62–7.52 (m, 1H), 7.52–7.40 (m, 2H), 5.76 (dd, *J* = 8.4, 5.9 Hz, 1H), 3.09–2.90 (m, 2H), 2.55–2.43 (m, 1H), 2.34–2.24 (m, 1H), 1.93–1.76 (m, 2H), 1.70–1.63 (m, 1H), 0.99 (m, 3H). ^13^C NMR (126 MHz, CDCl3) δ 199.8, 136.9, 133.1, 128.6, 128.0, 52.6, 44.4, 35.8, 32.4, 30.1, 18.6. HRMS *m*/*z*: calcd for C_13_H_17_Br_2_O (M + H^+^) = 346.9641, found 346.9645.

*6,6-dibromo-4-ethyl-1-phenylhexan-1-one* (**2i**). Yellow viscous oil (60.1 mg, 83% yield). ^1^H NMR (400 MHz, Chloroform-d) δ 7.99–7.87 (m, 2H), 7.61–7.51 (m, 1H), 7.52–7.42 (m, 2H), 5.76 (t, *J* = 7.0 Hz, 1H), 3.04–2.91 (m, 2H), 2.48–2.33 (m, 2H), 1.82–1.64 (m, 3H), 1.48–1.33 (m, 2H), 0.93 (t, *J* = 7.4 Hz, 3H). ^13^C NMR (126 MHz, CDCl3) δ 199.9, 136.9, 133.1, 128.6, 128.0, 49.8, 44.8, 38.2, 35.5, 26.4, 25.1, 10.3. HRMS *m*/*z*: calcd for C_14_H_19_Br_2_O (M + H^+^) = 360.9797, found 360.9799.

*6,6-dibromo-4,4-difluoro-1-phenylhexan-1-one* (**2j**). Yellow viscous oil (53.3 mg, 72% yield). ^1^H NMR (400 MHz, Chloroform-d) δ 8.02–7.92 (m, 2H), 7.65–7.54 (m, 1H), 7.55–7.42 (m, 2H), 5.89 (t, *J* = 6.4 Hz, 1H), 3.29–3.11 (m, 4H), 2.48–2.29 (m, 2H). ^13^C NMR (126 MHz, CDCl3) δ 197.6, 136.4, 133.5, 128.7, 128.0, 124.3, 122.4, 120.4, 52.1, 51.9, 51.7, 33.0, 32.9, 32.9, 31.2, 31.1, 30.9, 30.8, 30.8, 30.7. HRMS *m*/*z*: calcd for C_12_H_13_Br_2_F_2_O (M + H^+^) = 368.9296, found 368.9300.

*5,5-dibromo-1-phenylpentan-1-one* (**7a**). Yellow viscous oil (51.2 mg, 80% yield). ^1^H NMR (500 MHz, Chloroform-d) δ 7.99–7.88 (m, 2H), 7.65–7.51 (m, 1H), 7.53–7.38 (m, 2H), 5.76 (t, *J* = 6.2 Hz, 1H), 3.06 (t, *J* = 7.1 Hz, 2H), 2.62–2.41 (m, 2H), 2.04–1.96 (m, 2H). ^13^C NMR (126 MHz, CDCl3) δ 199.0, 136.7, 133.2, 128.7, 128.0, 45.3, 44.6, 36.8, 22.6. HRMS *m*/*z*: calcd for C_11_H_13_Br_2_O (M + H^+^) = 318.9328, found 318.9331.

*5,5-dibromo-1-(4-(trifluoromethyl)phenyl)pentan-1-one* (**7b**). Yellow viscous oil (61.3 mg, 79% yield). ^1^H NMR (400 MHz, Chloroform-d) δ 8.17–7.90 (m, 2H), 7.74 (d, *J* = 8.2 Hz, 2H), 5.77 (t, *J* = 6.1 Hz, 1H), 3.09 (t, *J* = 7.0 Hz, 2H), 2.57–2.46 (m, 2H), 2.09–1.93 (m, 2H). ^13^C NMR (126 MHz, CDCl3) δ 197.9, 139.3, 134.7, 134.4, 128.3, 125.8, 125.8, 124.6, 122.5, 45.0, 44.4, 37.2, 22.4. HRMS *m*/*z*: calcd for C_12_H_12_Br_2_F_3_O (M + H^+^) = 386.9202, found 386.9204.

*5,5-dibromo-1-(3-(tert-butyl)phenyl)pentan-1-one* (**7c**). Yellow viscous oil (54.2 mg, 72% yield). ^1^H NMR (400 MHz, Chloroform-d) δ 8.05–7.94 (m, 1H), 7.79–7.67 (m, 1H), 7.67–7.56 (m, 1H), 7.46–7.34 (m, 1H), 5.76 (t, *J* = 6.2 Hz, 1H), 3.06 (t, *J* = 7.0 Hz, 2H), 2.61–2.45 (m, 2H), 2.10–1.93 (m, 2H), 1.36 (s, 9H). ^13^C NMR (126 MHz, CDCl3) δ 199.3, 151.8, 136.5, 130.4, 128.4, 125.4, 124.7, 45.4, 44.7, 36.9, 34.9, 31.3, 22.7. HRMS *m*/*z*: calcd for C_15_H_20_Br_2_ONa (M + Na^+^) = 396.9773, found 396.9772.

*5,5-dibromo-1-(3-fluorophenyl)pentan-1-one* (**7d**). Yellow viscous oil (46.6 mg, 69% yield). ^1^H NMR (400 MHz, Chloroform-d) δ 7.76–7.69 (m, 1H), 7.67–7.59 (m, 1H), 7.51–7.43 (m, 1H), 7.31–7.25 (m, 1H), 5.76 (t, *J* = 6.1 Hz, 1H), 3.04 (t, *J* = 7.1 Hz, 2H), 2.55–2.43 (m, 2H), 2.05–1.92 (m, 2H). ^13^C NMR (126 MHz, CDCl3) δ 197.7, 163.9, 161.9, 138.8, 138.7, 130.4, 130.3, 123.7, 123.7, 120.4, 120.2, 114.9, 114.7, 45.1, 44.5, 37.0, 22.5. HRMS *m*/*z*: calcd for C_11_H_12_Br_2_FO (M + H^+^) = 336.9233, found 336.9229.

*Benzophenone* (**8a**). White solid (24.4 mg, 67% yield). ^1^H NMR (400 MHz, Chloroform-d) δ 7.91–7.69 (m, 4H), 7.59–7.57 (m, 2H), 7.48 (t, *J* = 7.6 Hz, 4H). The spectroscopic data are in accordance with the reported data [53].

*Acetophenone* (**8b**). Colorless oil (9.9 mg, 41% yield). ^1^H NMR (400 MHz, Chloroform-d) δ 7.95–7.91 (m, 2H), 7.55–7.53 (m, 1H), 7.53–7.41 (m, 2H), 2.57 (s, 3H). The spectroscopic data are in accordance with the reported data [54].

## 4. Conclusions

In summary, we have developed an EDA complex approach for preparing geminal dibromides from alkenes under mild conditions using visible light. Mechanistic studies suggest that the hypervalent iodine intermediate BI-OR, reacting as the electron donor for single-electron transfer, is crucial to the success of the reaction.

## Figures and Tables

**Table 1:**
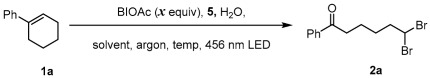
Optimization of the ring-opening geminal dibromination ^1^.

Entry	x	Solvent	Temperature	Yield(%)
1	1	MeCN	r.t.	25
2	1.5	MeCN	r.t.	39
3	2	MeCN	r.t.	54
4 ^2^	2	MeCN	r.t.	52
5	2	MeCN	50	76
6	2	DCM	50	46
7	2	PhCl	50	8
8	2	Acetone	50	ND
9	2	THF	50	ND
10	2	EtOAc	50	24
11 ^3^	2	MeCN	50	83
12 ^4^	2	MeCN	50	ND

^1^ Reaction conditions: **1a** (0.2 mmol), **5** (0.44 mmol), H_2_O (10 mmol), solvent (2 mL), under argon, 24 h. ^2^ NBS (0.44 mmol) was used instead of **5**. ^3^ Reaction time was 36 h. ^4^ No light irradiation.

## Data Availability

The data presented in this study are available in the main article or Supplementary Materials here.

## Supplementary Materials

The following supporting information can be downloaded at https://www.mdpi.com/article/10.3390/molecules29143281/s1. The following are available online: Figure S1: The charge-transfer bands between **3** and **4**/**5**; Figure S2: Emission profile of the Kessil^®^ PR160L@456 nm used to irradiate the reaction mixture; Figure S3: The charge-transfer bands between **3** and NCS/PhthN-Cl; Figure S4: Job plots of the EDA complexes between **3** and **5**; Figure S5: NMR studies of the EDA complexes between **3** and **5**; Figure S6: Light on–off experiment; Figures S7–S23: NMR spectra. References [55,56,57] are cited in Supplementary Materials.

## References

[1] Rosokha S.V., Kochi J.K. (2008). Fresh Look at Electron-Transfer Mechanisms via the Donor/Acceptor Bindings in the Critical Encounter Complex. Acc. Chem. Res..

[2] Foster R. (1980). Electron donor-acceptor complexes. J. Phys. Chem..

[3] Crisenza G.E.M., Mazzarella D., Melchiorre P. (2020). Synthetic Methods Driven by the Photoactivity of Electron Donor–Acceptor Complexes. J. Am. Chem. Soc..

[4] Yuan Y.-Q., Majumder S., Yang M.-H., Guo S.-R. (2020). Recent advances in catalyst-free photochemical reactions via electron-donor-acceptor (EDA) complex process. Tetrahedron Lett..

[5] Silvi M., Melchiorre P. (2018). Enhancing the potential of enantioselective organocatalysis with light. Nature.

[6] Lima C.G.S., de MLima T., Duarte M., Jurberg I.D., Paixão M.W. (2016). Organic Synthesis Enabled by Light-Irradiation of EDA Complexes: Theoretical Background and Synthetic Applications. ACS Catal..

[7] Arceo E., Jurberg I.D., Álvarez-Fernández A., Melchiorre P. (2013). Photochemical activity of a key donor–acceptor complex can drive stereoselective catalytic α-alkylation of aldehydes. Nat. Chem..

[8] Murphy J.J., Bastida D., Paria S., Fagnoni M., Melchiorre P. (2016). Asymmetric catalytic formation of quaternary carbons by iminium ion trapping of radicals. Nature.

[9] Zhang J., Li Y., Xu R., Chen Y. (2017). Donor–Acceptor Complex Enables Alkoxyl Radical Generation for Metal-Free C(sp3)–C(sp3) Cleavage and Allylation/Alkenylation. Angew. Chem. Int. Ed..

[10] Li Y., Zhang J., Li D., Chen Y. (2018). Metal-Free C(sp3)–H Allylation via Aryl Carboxyl Radicals Enabled by Donor–Acceptor Complex. Org. Lett..

[11] Davies J., Booth S.G., Essafi S., Dryfe R.A.W., Leonori D. (2015). Visible-Light-Mediated Generation of Nitrogen-Centered Radicals: Metal-Free Hydroimination and Iminohydroxylation Cyclization Reactions. Angew. Chem. Int. Ed..

[12] Quint V., Morlet-Savary F., Lohier J.-F., Lalevée J., Gaumont A.-C., Lakhdar S. (2016). Metal-Free, Visible Light-Photocatalyzed Synthesis of Benzo[b]phosphole Oxides: Synthetic and Mechanistic Investigations. J. Am. Chem. Soc..

[13] Cheng Y., Yu S. (2016). Hydrotrifluoromethylation of Unactivated Alkenes and Alkynes Enabled by an Electron-Donor–Acceptor Complex of Togni’s Reagent with a Tertiary Amine. Org. Lett..

[14] Fawcett A., Pradeilles J., Wang Y., Mutsuga T., Myers E.L., Aggarwal V.K. (2017). Photoinduced decarboxylative borylation of carboxylic acids. Science.

[15] Spell M.L., Deveaux K., Bresnahan C.G., Bernard B.L., Sheffield W., Kumar R., Ragains J.R. (2016). A Visible-Light-Promoted O-Glycosylation with a Thioglycoside Donor. Angew. Chem. Int. Ed..

[16] Sandfort F., Strieth-Kalthoff F., Klauck F.J.R., James M.J., Glorius F. (2018). Deaminative Borylation of Aliphatic Amines Enabled by Visible Light Excitation of an Electron Donor–Acceptor Complex. Chem. Eur. J..

[17] Sun J., He Y., An X.-D., Zhang X., Yu L., Yu S. (2018). Visible-light-induced iminyl radical formation via electron-donor–acceptor complexes: A photocatalyst-free approach to phenanthridines and quinolines. Org. Chem. Front..

[18] Gray P., Williams A. (1959). The Thermochemistry and Reactivity Of Alkoxyl Radicals. Chem. Rev..

[19] Batt L. (1987). Reactions of alkoxy and alkyl peroxy radicals. Int. Rev. Phy. Chem..

[20] Ren R., Zhu C. (2016). Radical-Mediated Ring-Opening Functionalization of Cyclobutanols: A Shortcut to γ-Substituted Ketones. Synlett.

[21] Tsui E., Wang H., Knowles R.R. (2020). Catalytic generation of alkoxy radicals from unfunctionalized alcohols. Chem. Sci..

[22] Concepción J.I., Francisco C.G., Hernández R., Salazar J.A., Suárez E. (1984). Intramolecular hydrogen abstraction. Iodosobenzene diacetate, an efficient and convenient reagent for alkoxy radical generation. Tetrahedron Lett..

[23] Lee J., Oh J., Jin S.-J., Choi J.-R., Atwood J.L., Cha J.K. (1994). Fragmentation of Alkoxy Radicals and Oxidative Elimination of Alicyclic Iodides. J. Org. Chem..

[24] Zhao P., Incarvito C.D., Hartwig J.F. (2006). Direct Observation of β-Aryl Eliminations from Rh(I) Alkoxides. J. Am. Chem. Soc..

[25] Ilangovan A., Saravanakumar S., Malayappasamy S. (2013). γ-Carbonyl Quinones: Radical Strategy for the Synthesis of Evelynin and Its Analogues by C–H Activation of Quinones Using Cyclopropanols. Org. Lett..

[26] Zhao H., Fan X., Yu J., Zhu C. (2015). Silver-Catalyzed Ring-Opening Strategy for the Synthesis of β- and γ-Fluorinated Ketones. J. Am. Chem. Soc..

[27] Lu S.-C., Li H.-S., Xu S., Duan G.-Y. (2017). Silver-catalyzed C2-selective direct alkylation of heteroarenes with tertiary cycloalkanols. Org. Biomol. Chem..

[28] Yamamoto K., Toguchi H., Kuriyama M., Watanabe S., Iwasaki F., Onomura O. (2021). Electrophotochemical Ring-Opening Bromination of tert-Cycloalkanols. J. Org. Chem..

[29] Chang L., An Q., Duan L., Feng K., Zuo Z. (2022). Alkoxy Radicals See the Light: New Paradigms of Photochemical Synthesis. Chem. Rev..

[30] Hu A., Guo J.-J., Pan H., Zuo Z. (2018). Selective functionalization of methane, ethane, and higher alkanes by cerium photocatalysis. Science.

[31] Guo J.-J., Hu A., Chen Y., Sun J., Tang H., Zuo Z. (2016). Photocatalytic C–C Bond Cleavage and Amination of Cycloalkanols by Cerium(III) Chloride Complex. Angew. Chem. Int. Ed..

[32] Jia K., Chen Y. (2018). Visible-light-induced alkoxyl radical generation for inert chemical bond cleavage/functionalization. Chem. Commun..

[33] Zhang J., Li Y., Zhang F., Hu C., Chen Y. (2016). Generation of Alkoxyl Radicals by Photoredox Catalysis Enables Selective C(sp3)–H Functionalization under Mild Reaction Conditions. Angew. Chem. Int. Ed..

[34] Zhang J., Liu D., Liu S., Ge Y., Lan Y., Chen Y. (2020). Visible-Light-Induced Alkoxyl Radicals Enable α-C(sp3)-H Bond Allylation. iScience.

[35] Ge Y., Shao Y., Wu S., Liu P., Li J., Qin H., Zhang Y., Xue X.-S., Chen Y. (2023). Distal Amidoketone Synthesis Enabled by Dimethyl Benziodoxoles via Dual Copper/Photoredox Catalysis. ACS Catal..

[36] Cao Z., Wang X., Wu X., Zhu C. (2022). Iodobenzene-catalyzed photochemical heteroarylation of alcohols by rupture of inert C–H and C–C bonds. Tetrahedron Chem..

[37] Wu X., Zhang H., Tang N., Wu Z., Wang D., Ji M., Xu Y., Wang M., Zhu C. (2018). Metal-free alcohol-directed regioselective heteroarylation of remote unactivated C(sp3)–H bonds. Nat. Commun..

[38] Wang C., Harms K., Meggers E. (2016). Catalytic Asymmetric C–H Functionalization under Photoredox Conditions by Radical Translocation and Stereocontrolled Alkene Addition. Angew. Chem. Int. Ed..

[39] Liang Z., Liu C., Fan J., Wang M., Yan X., Huang M., Cai S. (2022). Photocatalytic dehydrogenated etherification of 2-aryl benzylic alcohols. Green Chem..

[40] Hu X., Li G.-X., He G., Chen G. (2019). Minisci C–H alkylation of N-heteroarenes with aliphatic alcohols via β-scission of alkoxy radical intermediates. Org. Chem. Front..

[41] Li G.-X., Hu X., He G., Chen G. (2019). Photoredox-mediated remote C(sp3)–H heteroarylation of free alcohols. Chem. Sci..

[42] Shi J.-L., Wang Y., Wang Z., Dou B., Wang J. (2020). Ring-opening iodination and bromination of unstrained cycloalkanols through β-scission of alkoxy radicals. Chem. Commun..

[43] Rivero A.R., Fodran P., Ondrejková A., Wallentin C.-J. (2020). Alcohol Etherification via Alkoxy Radicals Generated by Visible-Light Photoredox Catalysis. Org. Lett..

[44] Murakami M., Ishida N. (2017). β-Scission of Alkoxy Radicals in Synthetic Transformations. Chem. Lett..

[45] Jia K., Pan Y., Chen Y. (2017). Selective Carbonyl–C(sp3) Bond Cleavage to Construct Ynamides, Ynoates, and Ynones by Photoredox Catalysis. Angew. Chem. Int. Ed..

[46] Jia K., Zhang F., Huang H., Chen Y. (2016). Visible-Light-Induced Alkoxyl Radical Generation Enables Selective C(sp3)–C(sp3) Bond Cleavage and Functionalizations. J. Am. Chem. Soc..

[47] Wu S., Chen Y. (2023). Selective C−H Acyloxylation of Sulfides/Disulfides Enabled by Hypervalent Iodine Reagents. Adv. Synth. Catal..

[48] Wu S., Li J., He R., Jia K., Chen Y. (2021). Terminal Trifluoromethylation of Ketones via Selective C–C Cleavage of Cycloalkanols Enabled by Hypervalent Iodine Reagents. Org. Lett..

[49] Prakash G.K.S., Mathew T., Hoole D., Esteves P.M., Wang Q., Rasul G., Olah G.A. (2004). N-Halosuccinimide/BF_3_–H_2_O, Efficient Electrophilic Halogenating Systems for Aromatics. J. Am. Chem. Soc..

[50] Bartoli S., Cipollone A., Squarcia A., Madami A., Fattori D. (2009). Electrophilic Bromination of meta-Substituted Anilines with N-Bromosuccin imide: Regioselectivity and Solvent Effect. Synthesis.

[51] Rogers D.A., Brown R.G., Brandeburg Z.C., Ko E.Y., Hopkins M.D., LeBlanc G., Lamar A.A. (2018). Organic Dye-Catalyzed, Visible-Light Photoredox Bromination of Arenes and Heteroarenes Using N-Bromosuccinimide. ACS Omega.

[52] Wang H., Toh R.W., Shi X., Wang T., Cong X., Wu J. (2020). Photo-mediated selective deconstructive geminal dihalogenation of trisubstituted alkenes. Nat. Commun..

[53] Narender M., Reddy M.S., Nageswar Y.V.D., Rao K.R. (2006). Aqueous phase synthesis of vic-halohydrins from olefins and N-halosuccinimides in the presence of β-cyclodextrin. J. Mol. Catal. A Chem..

[54] Inamoto K., Yamada T., Kato S., Kikkawa S., Kondo Y. (2013). Facile deprotection of dithioacetals by using a novel 1,4-benzoquinone/cat. NaI system. Tetrahedron.

[55] Yadav D.K., Lokhande R.S., Pitale S.M., Janwadkar S.P., Navarkar P.S., Rana P.K. (2014). Study of New Selective Reagent Acetophenone 2′, 4′-Dihydroxy Semicarbazone for Extractive Spectrophotometric Determination of Vanadium. World J. Anal. Chem..

[56] Jia K., Li J., Chen Y. (2018). Selective P−C(sp3) Bond Cleavage and Radical Alkynylation of α-Phosphorus Alcohols by Photoredox Catalysis. Chem. Eur. J..

[57] Kiyokawa K., Kosaka T., Kojima T., Minakata S. (2015). Synthesis and Structure of Hypervalent Iodine(III) Reagents Containing Phthalimidate and Application to Oxidative Amination Reactions. Angew. Chem. Int. Ed. Engl..

