# Supplementary material for: Visible-Light-Mediated Ring-Opening Geminal Dibromination of Alkenes via Alkoxy Radicals Enabled by Electron Donor–Acceptor Complex
Source: Molecules. 2024 Jul 11;29(14):3281. doi: 10.3390/molecules29143281 (PMC11278856; doi:10.3390/molecules29143281)
Supplement: Supplementary file 1 [file molecules-29-03281-s001.zip › molecules-3085694-supplementary.pdf]

Supporting Information

Visible-light Mediated Ring-Opening Geminal Dibromination of Alkenes via Alkoxy Radicals Enabled by Electron Donor-Acceptor Complex

Rong Wei [1], Yuan Wang [1,*], Juantao Zhang [1,*], Chunsheng Wu [2], Zhenhua Zhang [1] and Duo Zhang [3]

1 State Key Laboratory for Performance and Structure Safety of Petroleum Tubular Goods and Equipment Materials, Tubular Goods Research Institute of CNPC, Xi'an 710077, China; weir@cnpc.com.cn (R.W.); 202232655@stumail.nwu.edu.cn (Z.Z.)
2 National Engineering Laboratory of Low Permeability Oil-Gas Field Exploration and Development, Changqing Oilfield, Xi'an 710018, China; wucs01_cq@petrochina.com.cn
3 Medicine Center, Guangxi University of Science and Technology, Liuzhou 545006, China; duo.zhang@gxust.edu.cn
* Correspondence: wangyuan008@cnpc.com.cn (Y.W.); zhangjuantao@cnpc.com.cn (J.Z.)

Table of Contents

1. General Information S2
2. UV/Vis Absorption and NMR Studies S3
3. Ring-Opening Geminal Dibromination S6
4. Investigation of Other Trisubstituted Alkenes S9
5. Mechanistic Studies S11
6. References S13
7. NMR Spectra S14

1. General Information

Unless otherwise noted, all chemicals were purchased from commercial suppliers (Sigma Aldrich, TCI, Oakwood) and used without further purification. When required, solvents were dried according to general purification methods. The product mixtures were analyzed by thin layer chromatography using TLC silica gel plates (Merck-Schuchardt) with fluorescent indicator (λ = 254 nm). The purification of the products was performed by flash column chromatography using silica gel 60 (63-200 μm) from SANPONT. NMR spectra were recorded on Bruker AV-III400 (400 MHZ) or AMX500 (500 MHz) spectrometer in deuterated solvents. Chemical shifts (δ) are reported in parts per million (ppm) and spin-spin coupling constants (J) are given in Hz, while multiplicities are given the standard abbreviations: s (singlet), d (doublet), t (triplet), q (quartet), br (broad), m (multiplet). High resolution mass spectra (HRMS) were recorded on a Finnigan/MAT 95XL-T spectrometer. The diastereomeric ratio (dr) was determined by ^{1}H NMR of the crude product mixture. Absorption spectra were recorded in 1 cm path quartz cuvettes using an Edinburgh FS-5 spectrofluorometer. Continuous wave X-band ESR spectra were obtained with a JEOL (FA200) spectrometer. Trisubstituted alkenes, BI-OR reagent and bromohydrin were prepared following reported procedures.[s1-3]

2. UV/Vis Absorption and NMR Studies

Charge-transfer bands in optical absorption spectra

Optical absorption spectra between **3** (0.05 M) and **4/5** (0.05 M) in 1.0 mL MeCN were recorded recorded in 1 cm path quartz cuvettes using an Edinburgh FS-5 spectrofluorometer.

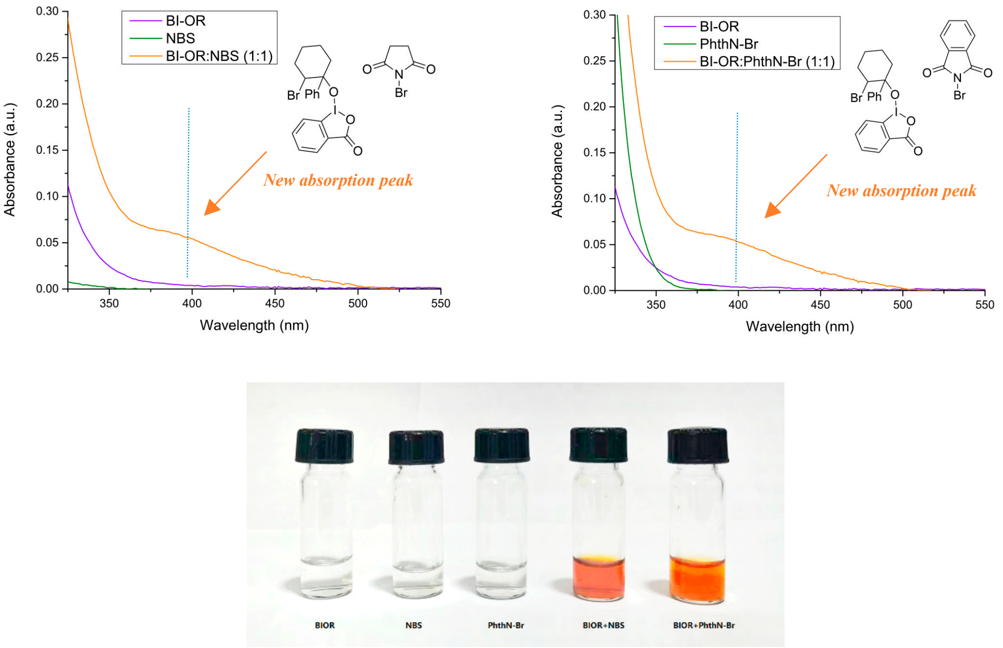

*Figure S1. The charge-transfer bands between **3** and **4/5***

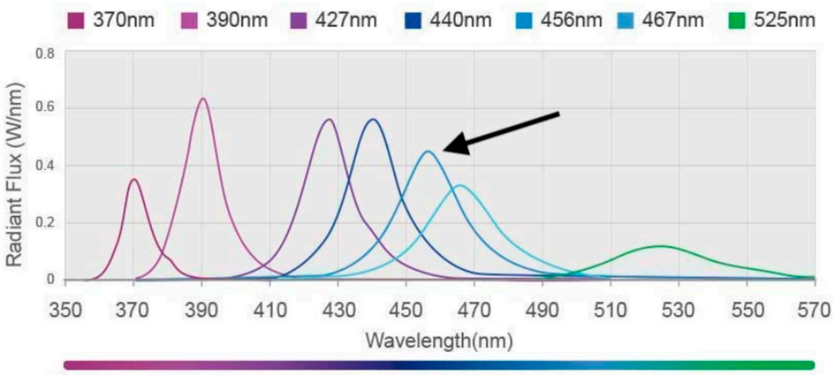

Figure S2. Emission profile of the Kessil® PR160L@456 nm used to irradiate the reaction mixture. (from Kessil® website https://www.kessil.com/science/PR160L.php).

From S1, above 400nm, there is almost no absorption in the local band, and the absorption is mainly due to the CT band. From S2, the wavelength of the light source is between 410-490nm, which does not overlap with the local band, but has a significant overlap with the CT band. This indicates that the excitation in the reaction is CT band excitation, not CT and local band excitation.

Optical absorption spectra between **3** (0.05 M) and **NCS/PhthNCl** (0.05 M) in 1.0 mL MeCN were recorded recorded in 1 cm path quartz cuvettes using an Edinburgh FS-5 spectrofluorometer.

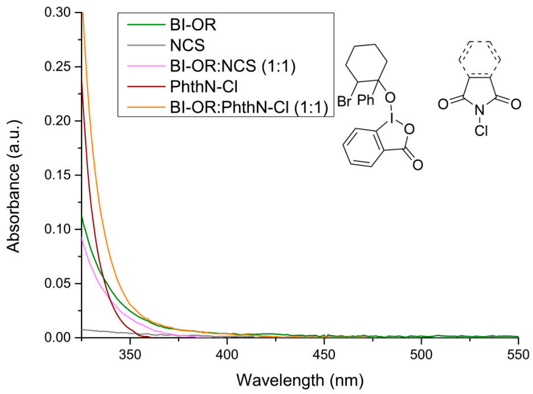

*Figure S3. The charge-transfer bands between **3** and NCS/PhthN-Cl*

The UV-vis results indicated that **3** could form EDA complex with **4** and **5**, but NCS or PhthNCl could not participate the formation of EDA complex.

Stoichiometry of the EDA Complex in Solution

The Job's plot was constructed to evaluate the stoichiometry of the EDA complex[s4] between **3** and **5**. We measured the absorption of DCM solutions at 445 nm with different donor/acceptor ratios with constant concentration (0.05 M) of the two components. All the absorption spectra were recorded in 1 cm path quartz cuvettes using an Edinburgh FS-5 spectrofluorometer. The absorbance values were plotted against the molar fraction (%) of **3**. The maximal absorbance at 50% molar fraction of **3** indicated the 1:1 stoichiometry of the EDA complex in solution.

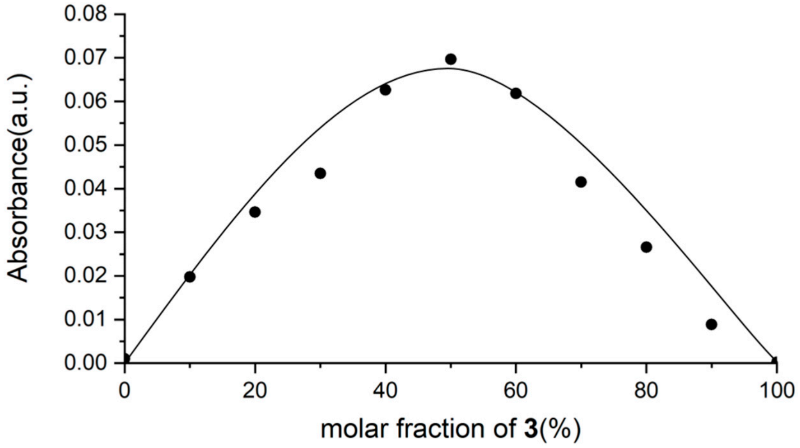

*Figure S4. Job's plots of the EDA complexes between **3** and **5***

NMR Experiments

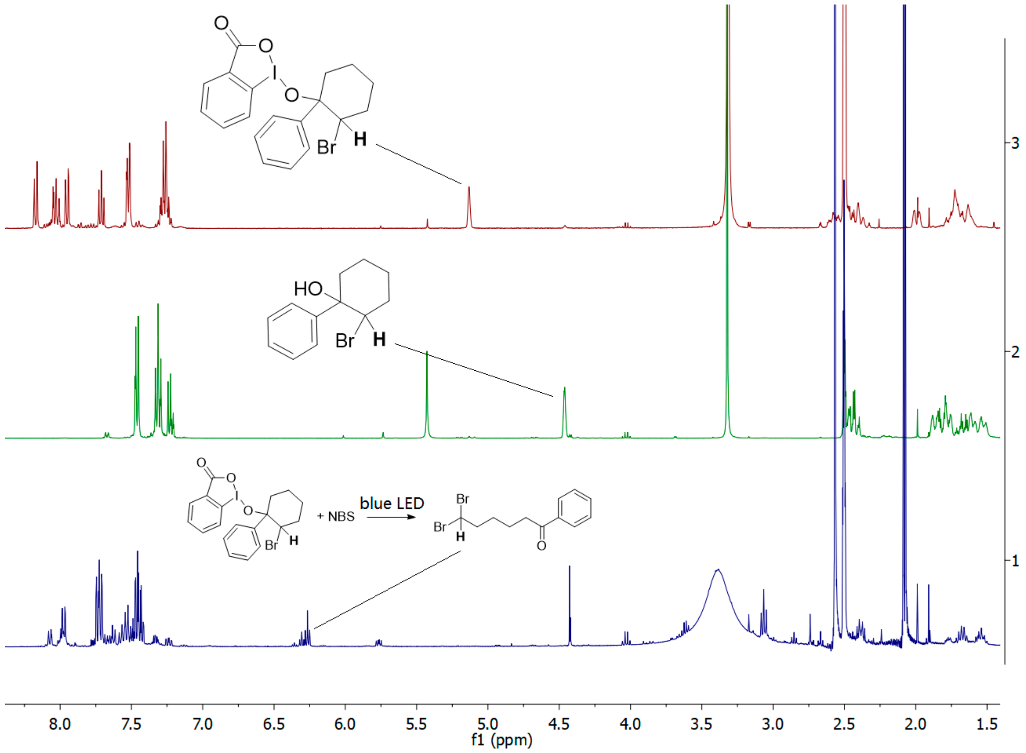

*Figure S5. NMR studies of the EDA complexes between **3** and **5***

Reaction design

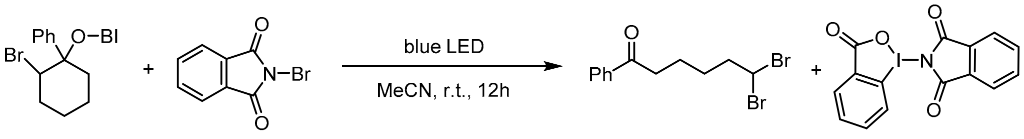

3 **5** **2a**, 66% **6**, 20%

3 (0.1 mmol, 1 equiv), **5** (0.1 mmol, 1 equiv), and MeCN (1 mL) were added to a schlenk tube (10 mL) equipped with a magnetic stirring bar. Then, the reaction mixture was operated by freeze-pump-thaw procedures for three times and backfilled with argon. The resulting solution was irradiated by blue LED lamps (2*40 W) and magnetically stirred. After 12 hours, the reaction solution was concentrated, and the product was purified by column chromatography (SiO2).

<u>Product **2a** was obtained as a yellow oil (22.0 mg, 66% yield).</u>

^{1}H NMR (400 MHz, Chloroform-*d*) δ 8.03 – 7.84 (m, 2H), 7.63 – 7.53 (m, 1H), 7.48 – 7.40 (m, 2H), 5.73 (t, *J* = 6.2 Hz, 1H), 3.02 (t, *J* = 7.2 Hz, 2H), 2.57 – 2.38 (m, 2H), 1.88 – 1.72 (m, 2H), 1.71 – 1.64 (m, 2H). ^{13}C NMR (126 MHz, $CDCl_3$) δ 199.7, 136.9, 133.1, 128.7, 128.1, 45.8, 45.3, 38.2, 27.9, 22.8. HRMS m/z: calcd for $C_{12}H_{14}Br_2ONa$ ($M+Na^+$) = 354.9304, found 354.9305.

<u>Product **6** was obtained as white soild (7.7 mg, 20% yield).</u>

^{1}H NMR (400 MHz, DMSO-d_6) δ 8.14 – 8.12 (m, 1H), 7.99 – 7.91 (m, 4H), 7.89 – 7.77 (m, 2H). ^{13}C NMR (101 MHz, DMSO-d_6) δ 171.5, 168.0, 136.6, 135.3, 133.3, 132.5, 131.9, 131.0, 127.6, 124.2, 119.4. The spectroscopic data are in accordance with the reported data.[s5]

Use of other light sources

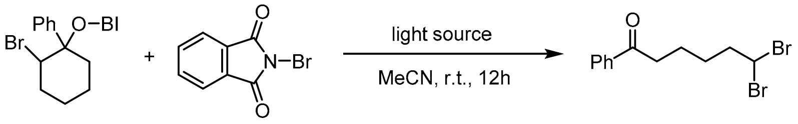

3 **5** **2**a

light source	yield (%)
Blue LED strips (456nm, 18W)	21
Blue LED lamps (456 nm, 40W X 2)	66
Green LED strips (520 nm, 18W)	trace
White LED strips (400-700 nm, 18W)	10

3. Ring-Opening Geminal Dibromination

3.1 General procedure

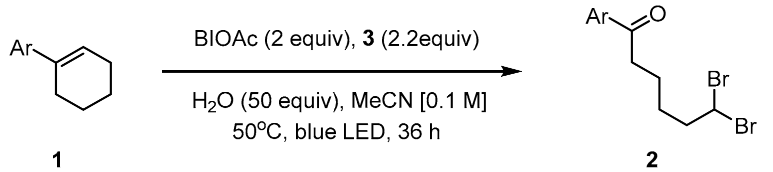

Alkene (0.2 mmol, 1.0 equiv), BIOAc (0.4 mmol, 2.0 equiv), **3** (0.44 mmol, 2.2 equiv), H_2O (10 mmol, 50.0 equiv), and MeCN (2.0 mL) were added to a schlenk tube (10 mL) equipped with a magnetic stirring bar. Then, the reaction mixture was operated by freeze-pump-thaw procedures for three times and backfilled with argon. The resulting solution was irradiated by blue LED lamps (2*40 W) and magnetically stirred at 50 ºC. After 36 hours, the reaction solution was concentrated, and the product was purified by column chromatography (SiO_2).

3.2 Photoreactor configuration

Reactions were irradiated using two 40W Kessil PR160 lamps (456 nm) while stirring on stir plates. The reactions were placed approximately 5 cm away from the LEDs (lux amount: about 150 mW/cm^2), and an external cooling fan was used to ensure that temperatures ranged from 48-52°C on average

Note: Intensity maps and spectral distribution of Kessil PR160L lamp could be found on website: https://kessil.com/products/science_PR160L.php

3.3 Characterization of products 2a-j

6,6-dibromo-1-phenylhexan-1-one (2a)

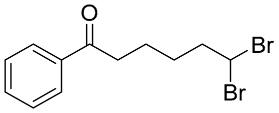

Following the general procedure afforded the product as a yellow viscous oil (55.5 mg, 83% yield). ^{1}H NMR (400 MHz, Chloroform-*d*) δ 8.03 – 7.84 (m, 2H), 7.63 – 7.53 (m, 1H), 7.48 – 7.40 (m, 2H), 5.73 (t, *J* = 6.2 Hz, 1H), 3.02 (t, *J* = 7.2 Hz, 2H), 2.57 – 2.38 (m, 2H), 1.88 – 1.72 (m, 2H), 1.71 – 1.64 (m, 2H). ^{13}C NMR (126 MHz, Chloroform-*d*) δ 199.7, 136.9, 133.1, 128.7, 128.1, 45.8, 45.3, 38.2, 27.9, 22.8. HRMS m/z: calcd for $C_{12}H_{14}Br_2ONa$ (M+Na$^+$) = 354.9304, found 354.9305.

6,6-dibromo-1-(4-methoxyphenyl)hexan-1-one (2b)

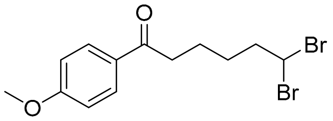

Following the general procedure afforded the product as a yellow viscous oil (43.7 mg, 60% yield). ^{1}H NMR (400 MHz, Chloroform-*d*) δ 7.94 (d, *J* = 8.9 Hz, 2H), 6.94 (d, *J* = 8.9 Hz, 2H), 5.72 (t, *J* = 6.2 Hz, 1H), 3.87 (s, 3H), 2.96 (t, *J* = 7.2 Hz, 2H), 2.56 – 2.31 (m, 2H), 1.90 – 1.71

(m, 2H), 1.71 – 1.58 (m, 2H). ^{13}C NMR (126 MHz, CDCl$_3$) δ 198.4, 163.6, 130.4, 130.2, 113.9, 55.6, 45.9, 45.4, 37.9, 28.0, 23.1. HRMS m/z: calcd for $C_{13}H_{16}Br_2O_2Na$ (M+Na$^+$) = 384.9409, found 384.9409.

6,6-dibromo-1-(4-(tert-butyl)phenyl)hexan-1-one (2c)

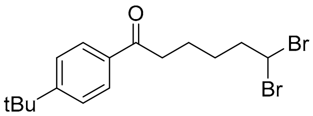

Following the general procedure afforded the product as a yellow viscous oil (67.1 mg, 86% yield). ^{1}H NMR (500 MHz, Chloroform-*d*) δ 7.99 – 7.73 (m, 2H), 7.62 – 7.42 (m, 2H), 5.73 (t, *J* = 6.2 Hz, 1H), 2.99 (t, *J* = 7.2 Hz, 2H), 2.58 – 2.29 (m, 2H), 1.92 – 1.68 (m, 2H), 1.69 – 1.56 (m, 2H), 1.34 (s, 9H). ^{13}C NMR (126 MHz, CDCl$_3$) δ 199.3, 156.8, 134.4, 128.0, 125.6, 125.5, 45.7, 45.2, 38.0, 35.1, 31.1, 27.8, 22.9. HRMS m/z: calcd for $C_{16}H_{12}Br_2ONa$ (M+Na$^+$) = 410.9930, found 410.9933.

1-([1,1'-biphenyl]-4-yl)-6,6-dibromohexan-1-one (2d)

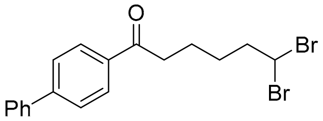

Following the general procedure afforded the product as a yellow viscous oil (68.9 mg, 84% yield). ^{1}H NMR (400 MHz, Chloroform-*d*) δ 8.07 – 8.00 (m, 2H), 7.71 – 7.56 (m, 4H), 7.51 – 7.45 (m, 2H), 7.42 – 7.36 (m, 1H), 5.74 (t, *J* = 6.2 Hz, 1H), 3.05 (t, *J* = 7.2 Hz, 2H), 2.53 – 2.42 (m, 2H), 1.87 – 1.79 (m, 2H), 1.73 – 1.61 (m, 2H). ^{13}C NMR (126 MHz, CDCl$_3$) δ 199.2, 145.8, 139.9, 135.6, 132.1, 129.0, 128.6, 128.3, 127.3, 45.7, 45.2, 38.2, 27.8, 22.8. HRMS m/z: calcd for $C_{18}H_{19}Br_2O$ (M+H$^+$) = 408.9797, found 408.9796.

6,6-dibromo-1-(4-fluorophenyl)hexan-1-one (2e)

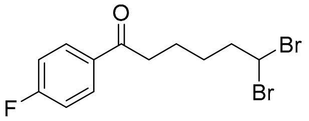

Following the general procedure afforded the product as a yellow viscous oil (50.0 mg, 71% yield). ^{1}H NMR (400 MHz, Chloroform-*d*) δ 8.05 – 7.93 (m, 2H), 7.18 – 7.08 (m, 2H), 5.73 (t, *J* = 6.1 Hz, 1H), 2.99 (t, *J* = 7.2 Hz, 2H), 2.50 – 2.36 (m, 2H), 1.87 – 1.71 (m, 2H), 1.70 – 1.62 (m, 2H). ^{13}C NMR (126 MHz, CDCl$_3$) δ 198.0, 166.8, 164.7, 133.3, 130.7, 130.6, 115.8, 115.6, 77.3, 77.0, 76.8, 45.6, 45.2, 38.0, 27.7, 22.7. HRMS m/z: calcd for $C_{12}H_{14}Br_2FO$ (M+H$^+$) = 350.9390, found 350.9390.

6,6-dibromo-1-(3-(tert-butyl)phenyl)hexan-1-one (2f)

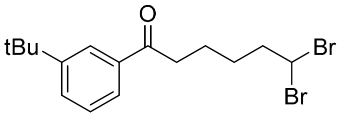

Following the general procedure afforded the product as a yellow viscous oil (57.7 mg, 74% yield). ^{1}H NMR (500 MHz, Chloroform-*d*) δ 8.05 – 7.92 (m, 1H), 7.81 – 7.71 (m, 1H), 7.65 – 7.55 (m, 1H), 7.46 – 7.33 (m, 1H), 5.73 (t, *J* = 6.1 Hz, 1H), 3.02 (t, *J* = 7.2 Hz, 2H), 2.46 (ddd,

J = 9.7, 7.0, 5.5 Hz, 2H), 1.90 – 1.76 (m, 2H), 1.72 – 1.61 (m, 2H), 1.36 (s, 9H). ^{13}C NMR (126 MHz, $CDCl_3$) δ 200.0, 151.8, 136.8, 130.2, 128.3, 125.4, 124.7, 45.7, 45.2, 38.2, 34.9, 31.3, 27.8, 22.8. HRMS m/z: calcd for $C_{16}H_{23}Br_2O$ (M+H$^+$) = 389.0110, found 389.0115.

6,6-dibromo-1-(3-fluorophenyl)hexan-1-one (2g)

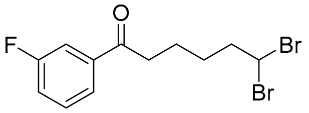

Following the general procedure afforded the product as a yellow viscous oil (52.8 mg, 75% yield). ^{1}H NMR (500 MHz, Chloroform-*d*) δ 7.75 – 7.71 (m, 1H), 7.66 – 7.60 (m, 1H), 7.49 – 7.40 (m, 1H), 7.31 – 7.23 (m, 1H), 5.73 (t, J = 6.1 Hz, 1H), 3.00 (t, J = 7.2 Hz, 2H), 2.48 – 2.40 (m, 2H), 1.81 (dt, J = 15.1, 7.3 Hz, 2H), 1.70 – 1.60 (m, 2H). ^{13}C NMR (126 MHz, $CDCl_3$) δ 198.3, 163.9, 161.9, 139.0, 138.9, 130.3, 130.3, 123.7, 123.7, 120.2, 120.0, 114.9, 114.7, 45.6, 45.1, 38.3, 27.7, 22.6. HRMS m/z: calcd for $C_{12}H_{14}Br_2FO$ (M+H$^+$) = 350.9390, found 350.9391.

6,6-dibromo-1-(4-methoxyphenyl)-4-methylhexan-1-one (2h)

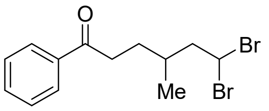

Following the general procedure afforded the product as a yellow viscous oil (57.8 mg, 83% yield). ^{1}H NMR (400 MHz, Chloroform-*d*) δ 8.04 – 7.90 (m, 2H), 7.62 – 7.52 (m, 1H), 7.52 – 7.40 (m, 2H), 5.76 (dd, J = 8.4, 5.9 Hz, 1H), 3.09 – 2.90 (m, 2H), 2.55 – 2.43 (m, 1H), 2.34 – 2.24 (m, 1H), 1.93 – 1.76 (m, 2H), 1.70 – 1.63 (m, 1H), 0.99 (m, 3H). ^{13}C NMR (126 MHz, $CDCl_3$) δ 199.8, 136.9, 133.1, 128.6, 128.0, 52.6, 44.4, 35.8, 32.4, 30.1, 18.6. HRMS m/z: calcd for $C_{13}H_{17}Br_2O$ (M+H$^+$) = 346.9641, found 346.9645.

6,6-dibromo-4-ethyl-1-phenylhexan-1-one (2i)

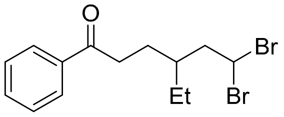

Following the general procedure afforded the product as a yellow viscous oil (60.1 mg, 83% yield). ^{1}H NMR (400 MHz, Chloroform-*d*) δ 7.99 – 7.87 (m, 2H), 7.61 – 7.51 (m, 1H), 7.52 – 7.42 (m, 2H), 5.76 (t, J = 7.0 Hz, 1H), 3.04 – 2.91 (m, 2H), 2.48 – 2.33 (m, 2H), 1.82 – 1.64 (m, 3H), 1.48 – 1.33 (m, 2H), 0.93 (t, J = 7.4 Hz, 3H). ^{13}C NMR (126 MHz, $CDCl_3$) δ 199.9, 136.9, 133.1, 128.6, 128.0, 49.8, 44.8, 38.2, 35.5, 26.4, 25.1, 10.3. HRMS m/z: calcd for $C_{14}H_{19}Br_2O$ (M+H$^+$) = 360.9797, found 360.9799.

6,6-dibromo-4,4-difluoro-1-phenylhexan-1-one (2j)

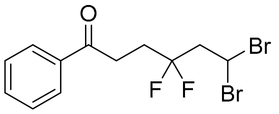

Following the general procedure afforded the product as a yellow viscous oil (53.3 mg, 72% yield). ^{1}H NMR (400 MHz, Chloroform-*d*) δ 8.02 – 7.92 (m, 2H), 7.65 – 7.54 (m, 1H), 7.55 – 7.42 (m, 2H), 5.89 (t, J = 6.4 Hz, 1H), 3.29 – 3.11 (m, 4H), 2.48 – 2.29 (m, 2H). ^{13}C NMR (126

MHz, $CDCl_3$) δ 197.6, 136.4, 133.5, 128.7, 128.0, 124.3, 122.4, 120.4, 52.1, 51.9, 51.7, 33.0, 32.9, 32.9, 31.2, 31.1, 30.9, 30.8, 30.8, 30.7. HRMS m/z: calcd for $C_{12}H_{13}Br_2F_2O$ ($M+H^+$) = 368.9296, found 368.9300.

4. Investigation of other trisubstituted alkenes

Cyclopentene derivatives

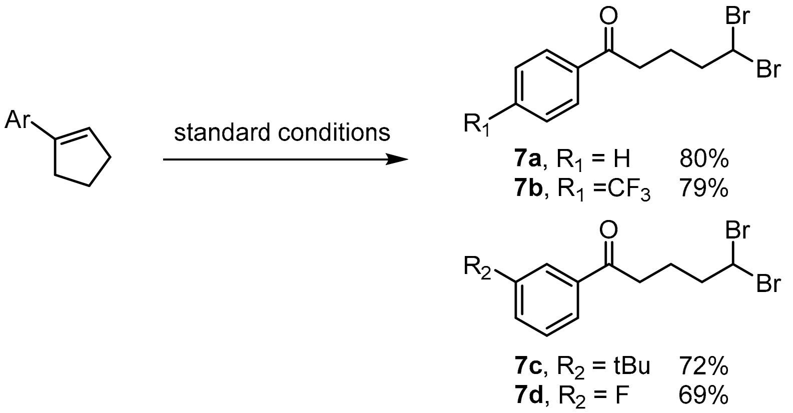

5,5-dibromo-1-phenylpentan-1-one (7a)

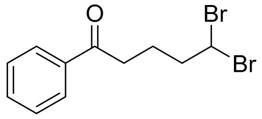

Following the general procedure afforded the product as a yellow viscous oil (51.2 mg, 80% yield). ^{1}H NMR (500 MHz, Chloroform-*d*) δ 7.99 – 7.88 (m, 2H), 7.65 – 7.51 (m, 1H), 7.53 – 7.38 (m, 2H), 5.76 (t, *J* = 6.2 Hz, 1H), 3.06 (t, *J* = 7.1 Hz, 2H), 2.62 – 2.41 (m, 2H), 2.04 – 1.96 (m, 2H). ^{13}C NMR (126 MHz, $CDCl_3$) δ 199.0, 136.7, 133.2, 128.7, 128.0, 45.3, 44.6, 36.8, 22.6. HRMS m/z: calcd for $C_{11}H_{13}Br_2O$ (M+H$^+$) = 318.9328, found 318.9331.

5,5-dibromo-1-(4-(trifluoromethyl)phenyl)pentan-1-one (7b)

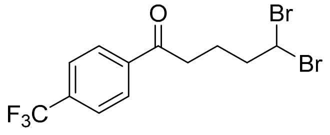

Following the general procedure afforded the product as a yellow viscous oil (61.3 mg, 79% yield). ^{1}H NMR (400 MHz, Chloroform-*d*) δ 8.17 – 7.90 (m, 2H), 7.74 (d, *J* = 8.2 Hz, 2H), 5.77 (t, *J* = 6.1 Hz, 1H), 3.09 (t, *J* = 7.0 Hz, 2H), 2.57 – 2.46 (m, 2H), 2.09 – 1.93 (m, 2H). ^{13}C NMR (126 MHz, $CDCl_3$) δ 197.9, 139.3, 134.7, 134.4, 128.3, 125.8, 125.8, 124.6, 122.5, 45.0, 44.4, 37.2, 22.4. HRMS m/z: calcd for $C_{12}H_{12}Br_2F_3O$ (M+H$^+$) = 386.9202, found 386.9204.

5,5-dibromo-1-(3-(tert-butyl)phenyl)pentan-1-one (7c)

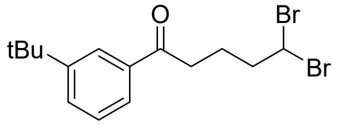

Following the general procedure afforded the product as a yellow viscous oil (54.2 mg, 72% yield). ^{1}H NMR (400 MHz, Chloroform-*d*) δ 8.05 – 7.94 (m, 1H), 7.79 – 7.67 (m, 1H), 7.67 – 7.56 (m, 1H), 7.46 – 7.34 (m, 1H), 5.76 (t, *J* = 6.2 Hz, 1H), 3.06 (t, *J* = 7.0 Hz, 2H), 2.61 – 2.45

(m, 2H), 2.10 – 1.93 (m, 2H), 1.36 (s, 9H). ^{13}C NMR (126 MHz, $CDCl_3$) δ 199.3, 151.8, 136.5, 130.4, 128.4, 125.4, 124.7, 45.4, 44.7, 36.9, 34.9, 31.3, 22.7. HRMS m/z: calcd for $C_{15}H_{20}Br_2ONa$ ($M+Na^+$) = 396.9773, found 396.9772.

5,5-dibromo-1-(3-fluorophenyl)pentan-1-one (7d)

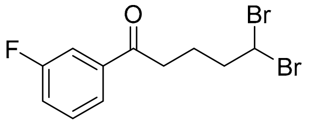

Following the general procedure afforded the product as a yellow viscous oil (46.6 mg, 69% yield). ^{1}H NMR (400 MHz, Chloroform-*d*) δ 7.76 – 7.69 (m, 1H), 7.67 – 7.59 (m, 1H), 7.51 – 7.43 (m, 1H), 7.31 – 7.25 (m, 1H), 5.76 (t, *J* = 6.1 Hz, 1H), 3.04 (t, *J* = 7.1 Hz, 2H), 2.55 – 2.43 (m, 2H), 2.05 – 1.92 (m, 2H). ^{13}C NMR (126 MHz, $CDCl_3$) δ 197.7, 163.9, 161.9, 138.8, 138.7, 130.4, 130.3, 123.7, 123.7, 120.4, 120.2, 114.9, 114.7, 45.1, 44.5, 37.0, 22.5. HRMS m/z: calcd for $C_{11}H_{12}Br_2FO$ ($M+H^+$) = 336.9233, found 336.9229.

Acyclic trisubstituted alkenes

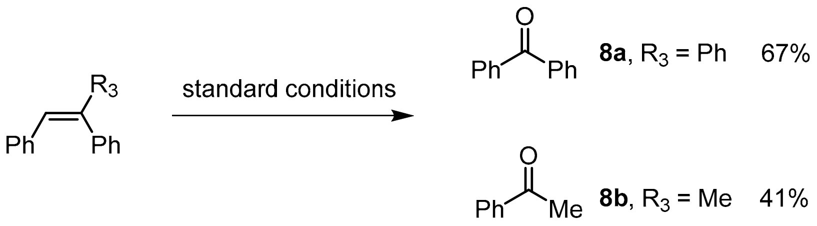

Benzophenone (8a)

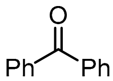

Following the general procedure afforded the product as a white solid (24.4 mg, 67% yield). ^{1}H NMR (400 MHz, Chloroform-*d*) δ 7.91 – 7.69 (m, 4H), 7.59– 7.57 (m, 2H), 7.48 (t, *J* = 7.6 Hz, 4H). The spectroscopic data are in accordance with the reported data.[s6]

Acetophenone (8b)

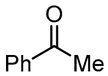

Following the general procedure afforded the product as a colourless oil (9.9 mg, 41% yield). ^{1}H NMR (400 MHz, Chloroform-*d*) δ 7.95 – 7.91 (m, 2H), 7.55 – 7.53 (m, 1H), 7.53 – 7.41 (m, 2H), 2.57 (s, 3H). The spectroscopic data are in accordance with the reported data.[s6]

5. Mechanistic Investigations

Control experiments

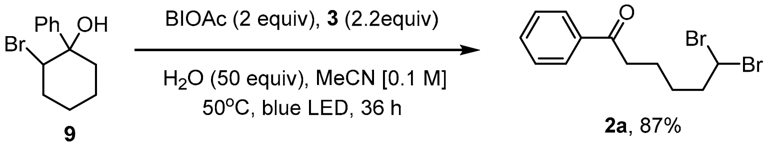

9 (0.2 mmol, 1.0 equiv), BIOAc (0.4 mmol, 2.0 equiv), **3** (0.44 mmol, 2.2 equiv), H_2O (10 mmol, 50.0 equiv), and MeCN (2.0 mL) were added to a schlenk tube (10 mL) equipped with a magnetic stirring bar. Then, the reaction mixture was operated by freeze-pump-thaw procedures for three times and backfilled with argon. The resulting solution was irradiated by blue LED lamps (2*40 W) and magnetically stirred at 50 °C. After 36 hours, the reaction solution was concentrated, and the product was purified by column chromatography (SiO_2).
Product **2a** was obtained as a yellow viscous oil (58.1 mg, 87%).

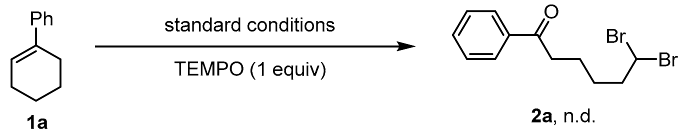

1a (0.2 mmol, 1.0 equiv), BIOAc (0.4 mmol, 2.0 equiv), **3** (0.44 mmol, 2.2 equiv), H_2O (10 mmol, 50.0 equiv), TEMPO (0.5 mmol, 2.5 equiv), and MeCN (2.0 mL) were added to a schlenk tube (10 mL) equipped with a magnetic stirring bar. Then, the reaction mixture was operated by freeze-pump-thaw procedures for three times and backfilled with argon. The resulting solution was irradiated by blue LED lamps (2*40 W) and magnetically stirred at 50 °C. After 36 hours, the reaction solution was concentrated, and the product was purified by column chromatography (SiO_2).
No product **2a** was detected.

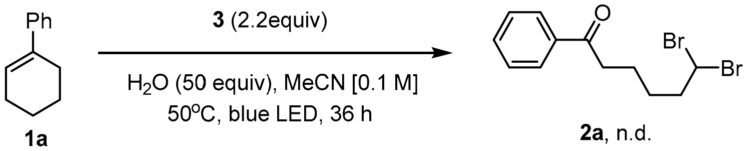

1a (0.2 mmol, 1.0 equiv), **3** (0.44 mmol, 2.2 equiv), H_2O (10 mmol, 50.0 equiv), and MeCN (2.0 mL) were added to a schlenk tube (10 mL) equipped with a magnetic stirring bar. Then, the reaction mixture was operated by freeze-pump-thaw procedures for three times and backfilled with argon. The resulting solution was irradiated by blue LED lamps (2*40 W) and magnetically stirred at 50 °C. After 36 hours, the reaction solution was concentrated, and the product was purified by column chromatography (SiO_2).
No product **2a** was detected.

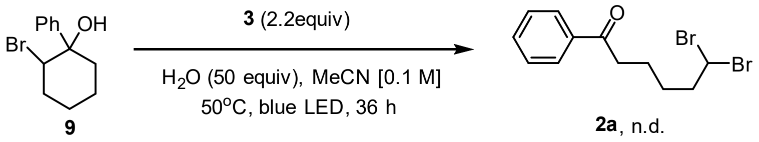

9 (0.2 mmol, 1.0 equiv), **3** (0.44 mmol, 2.2 equiv), H_2O (10 mmol, 50.0 equiv), and MeCN (2.0 mL) were added to a schlenk tube (10 mL) equipped with a magnetic stirring bar. Then, the reaction mixture was operated by freeze-pump-thaw procedures for three times and backfilled with argon. The resulting solution was irradiated by blue LED lamps (2*40 W) and magnetically stirred at 50 °C. After 36 hours, the reaction solution was concentrated, and the product was purified by column chromatography (SiO_2).

No product **2a** was detected.

Light on-off experiments

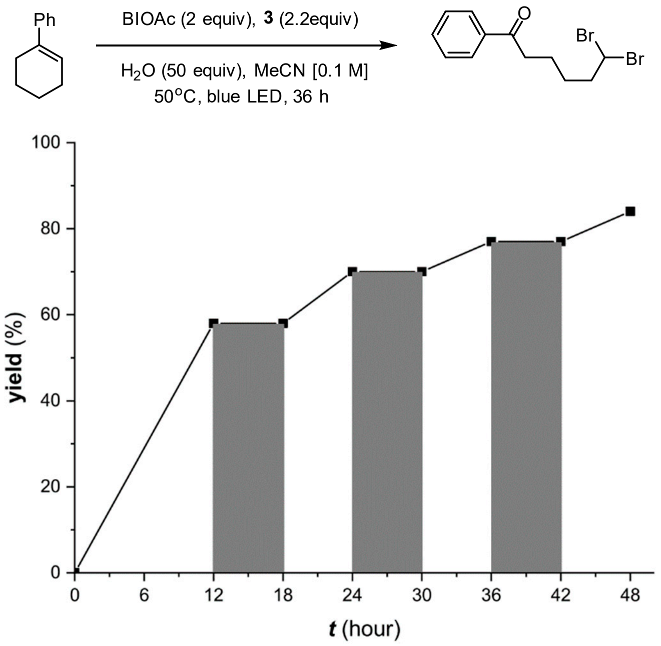

Figure S6. Light on-off experiment

Time profile of the transformation with the light ON/OFF over time. Yields were determined by crude ^{1}H NMR spectra using dibromomethane as an internal standard. To examine the impact of light, we conducted experiments under alternating periods of irradiation and darkness following the general procedure A. These resulted in total interruption of the reaction progress in the absence of light and recuperation of reactivity on further illumination. The results demonstrated that light was a necessary component of the reaction. Even though we could not fully rule out a radical-chain process, the data shown that any chain-propagation process must be short-lived.

6. References

[s1] K. Jia, F. Zhang, H. Huang, Y. Chen, *J. Am. Chem. Soc.* **2016**, 138, 1514.

[s2] H. Wang, R. W. Toh, X. Shi, T. Wang, X. Cong, J. Wu, *Nat Commun* **2020**, *11*, 4462.

[s3] K. Jia, J. Li, Y. Chen, *Chem. Eur. J.* **2018**, *24*, 3174.

[s4] P. Job, *Ann. Chim.* **1928**, *9*, 113.

[s5] K. Kiyokawa, T. Kosaka, T. Kojima, S. Minakata, *Angew. Chem., Int. Ed.* **2015**, *54*, 13719.

[s6] K. Inamoto, T. Yamada, Kato, S. Kikkawa, Y. Kondo, *Tetrahedron* **2013**, *44*, 9192.

7. NMR Spectra

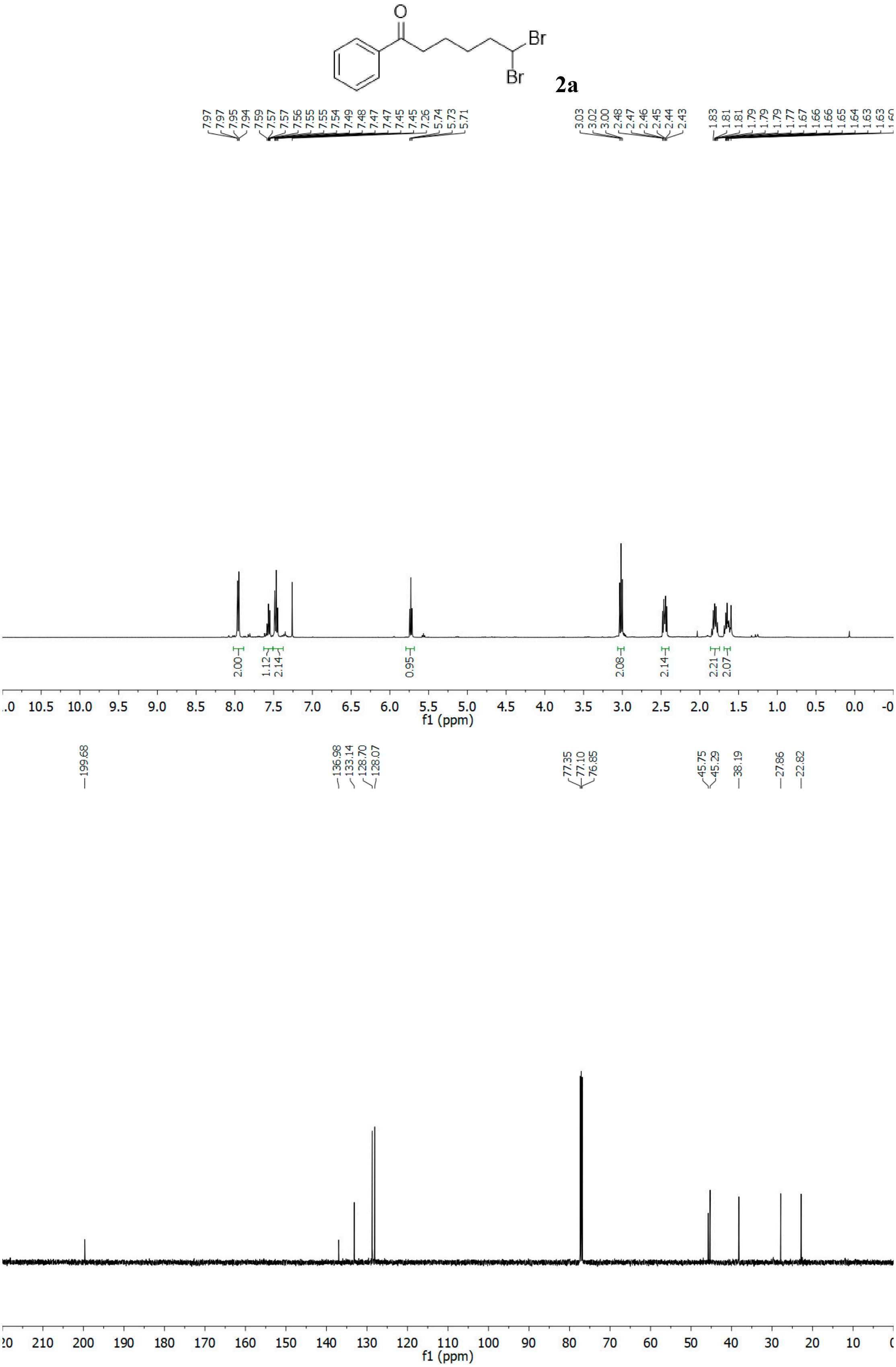

Figure S7. ^1H NMR spectrum ($CDCl_3$, 400 MHz) and ^{13}C NMR spectrum ($CDCl_3$, 101 MHz) of ***2a***

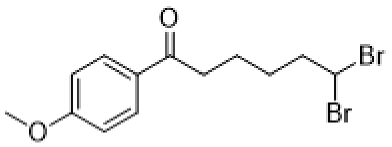

2b

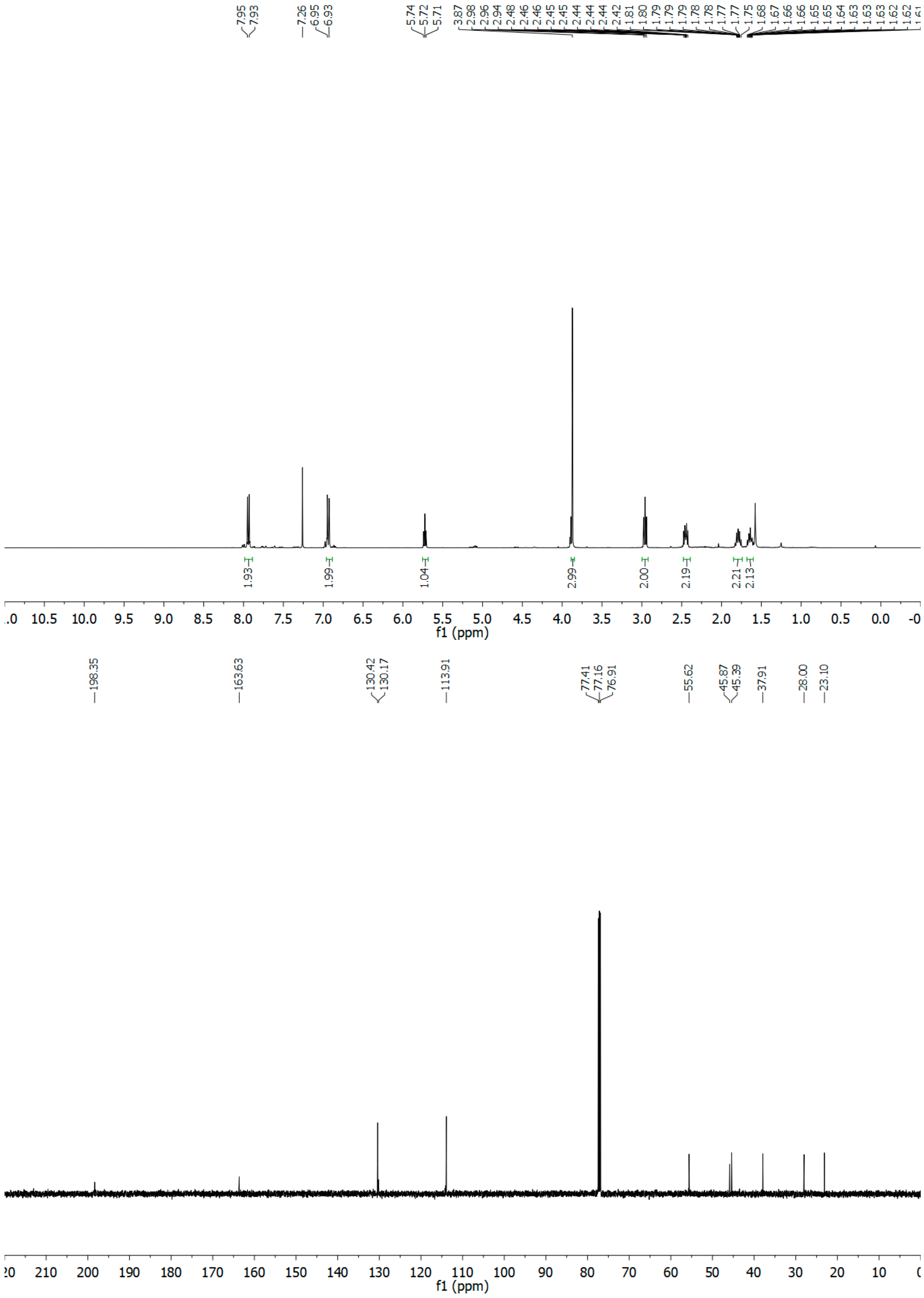

Figure S8. ^{1}H NMR spectrum ($CDCl_3$, 400 MHz) and ^{13}C NMR spectrum ($CDCl_3$, 101 MHz) of ***2b***

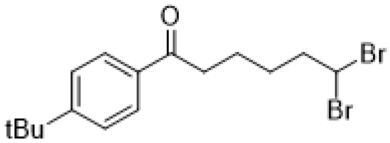

2c

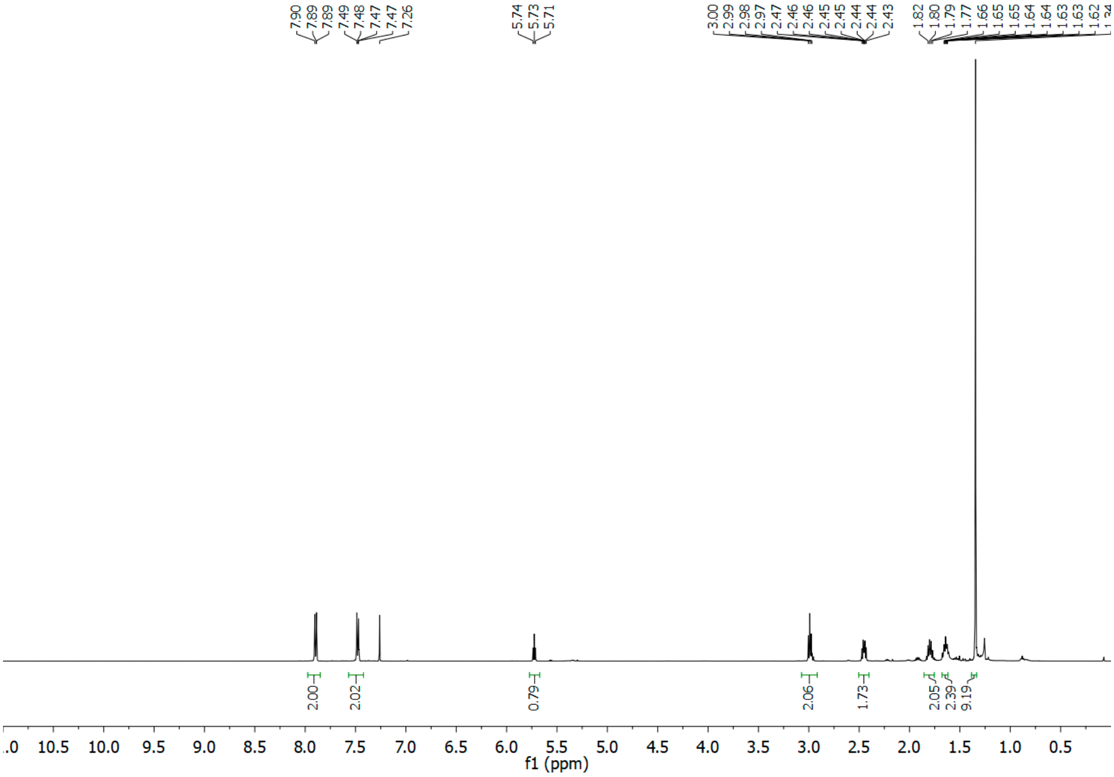

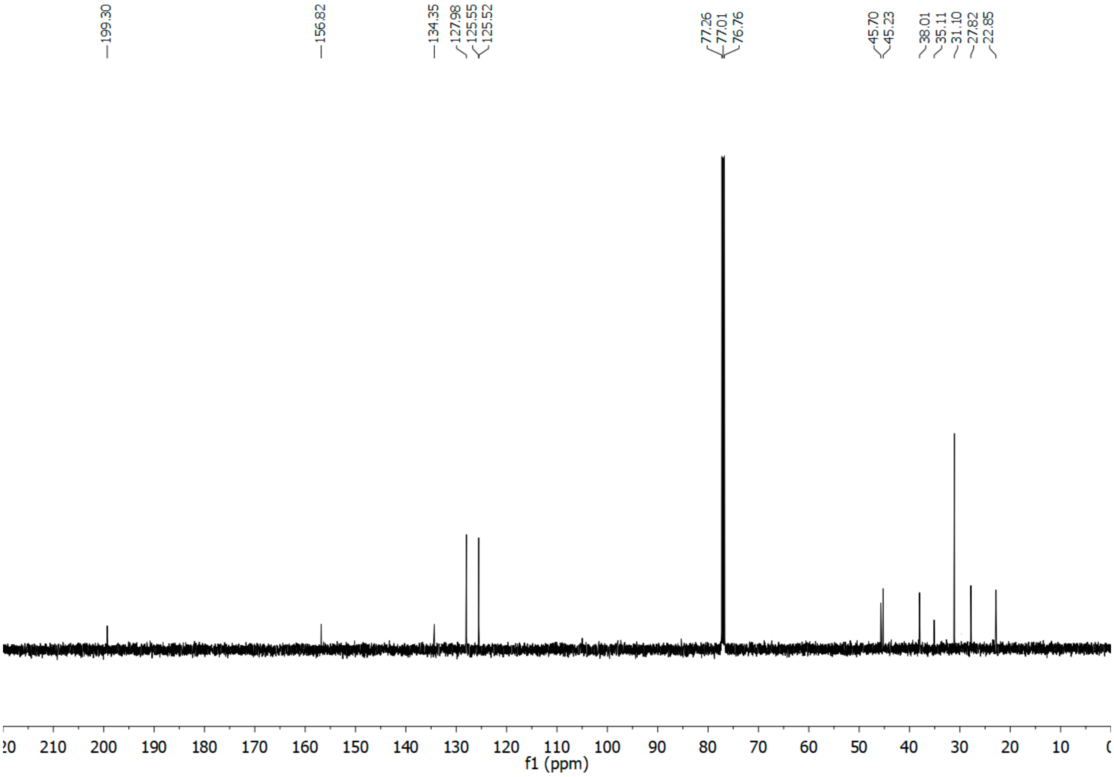

Figure S9. ^{1}H NMR spectrum (CDCl$_3$, 400 MHz) and ^{13}C NMR spectrum (CDCl$_3$, 101 MHz) of ***2c***

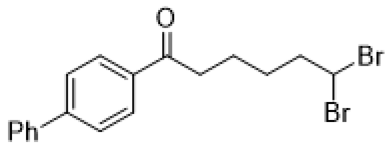

2d

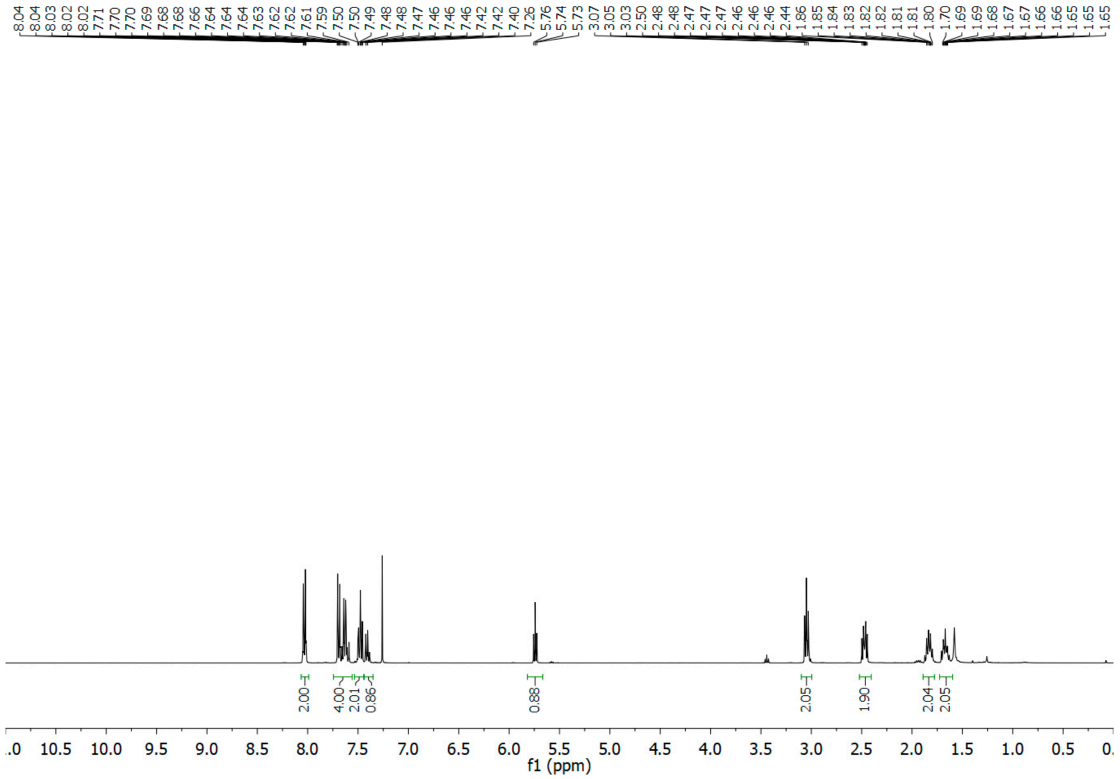

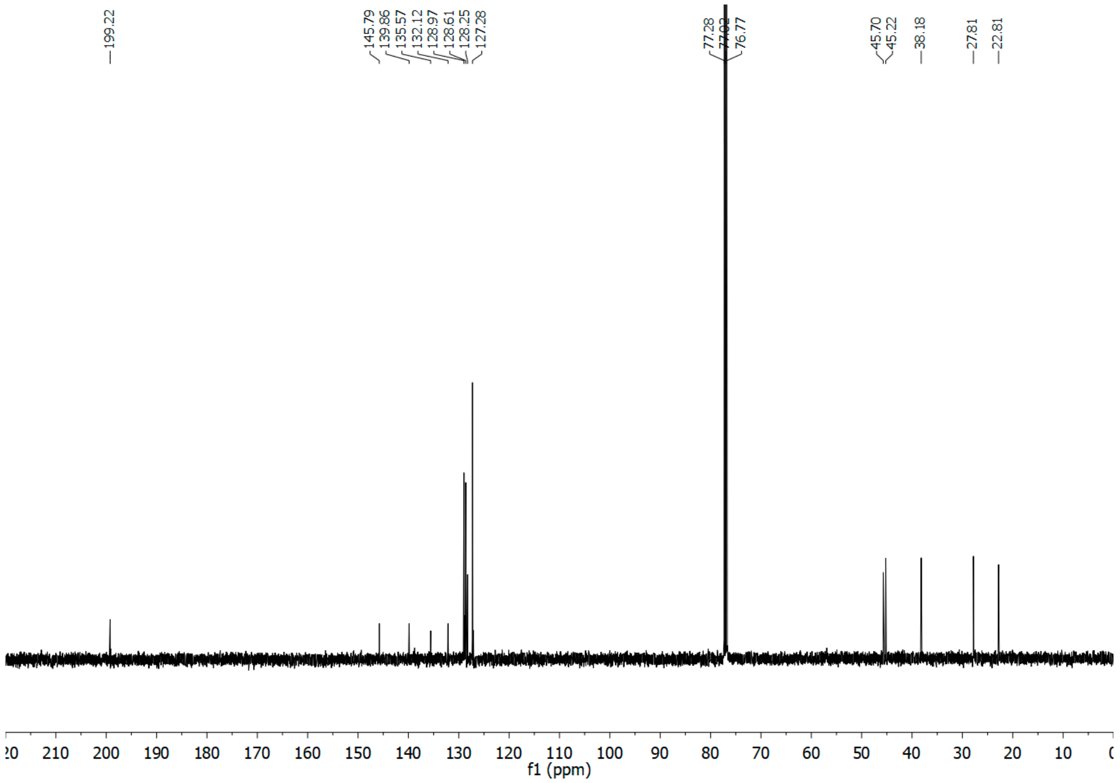

*Figure S10. ^{1}H NMR spectrum ($CDCl_3$, 400 MHz) and ^{13}C NMR spectrum ($CDCl_3$, 101 MHz) of **2d***

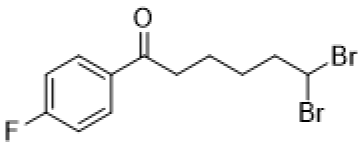

2e

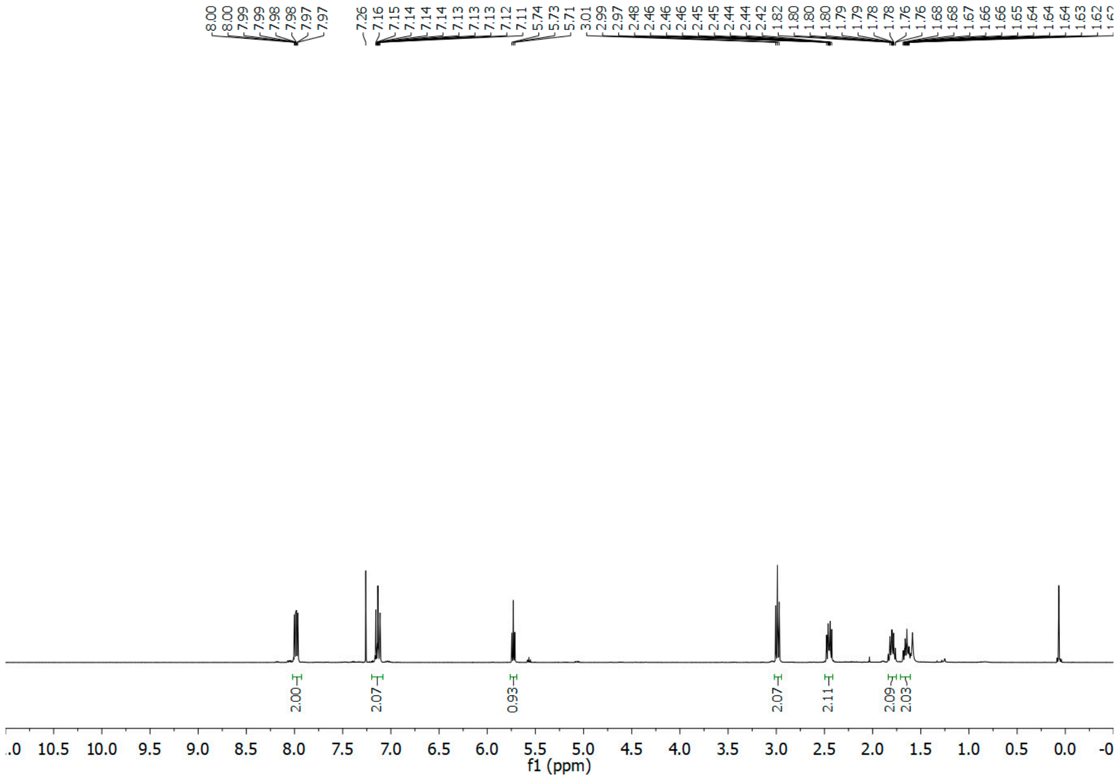

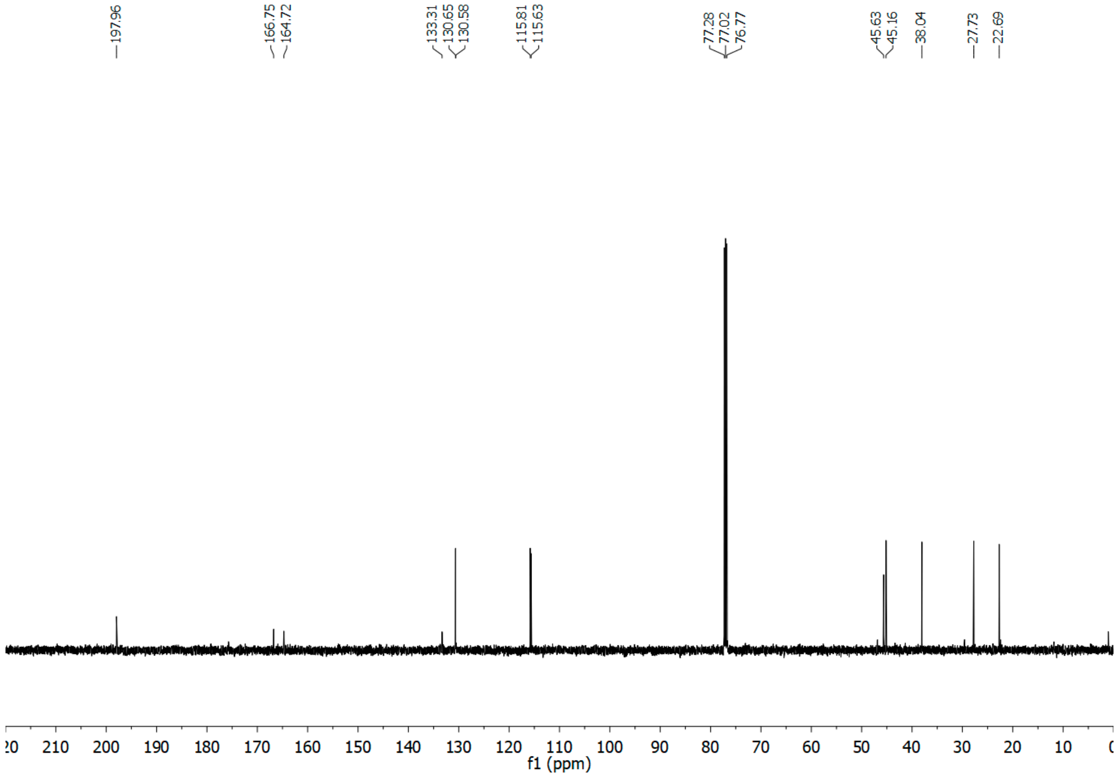

Figure S11. ^{1}H NMR spectrum ($CDCl_3$, 400 MHz) and ^{13}C NMR spectrum ($CDCl_3$, 101 MHz) of ***2e***

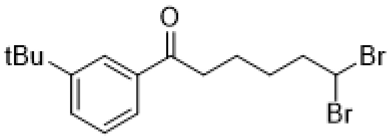

2f

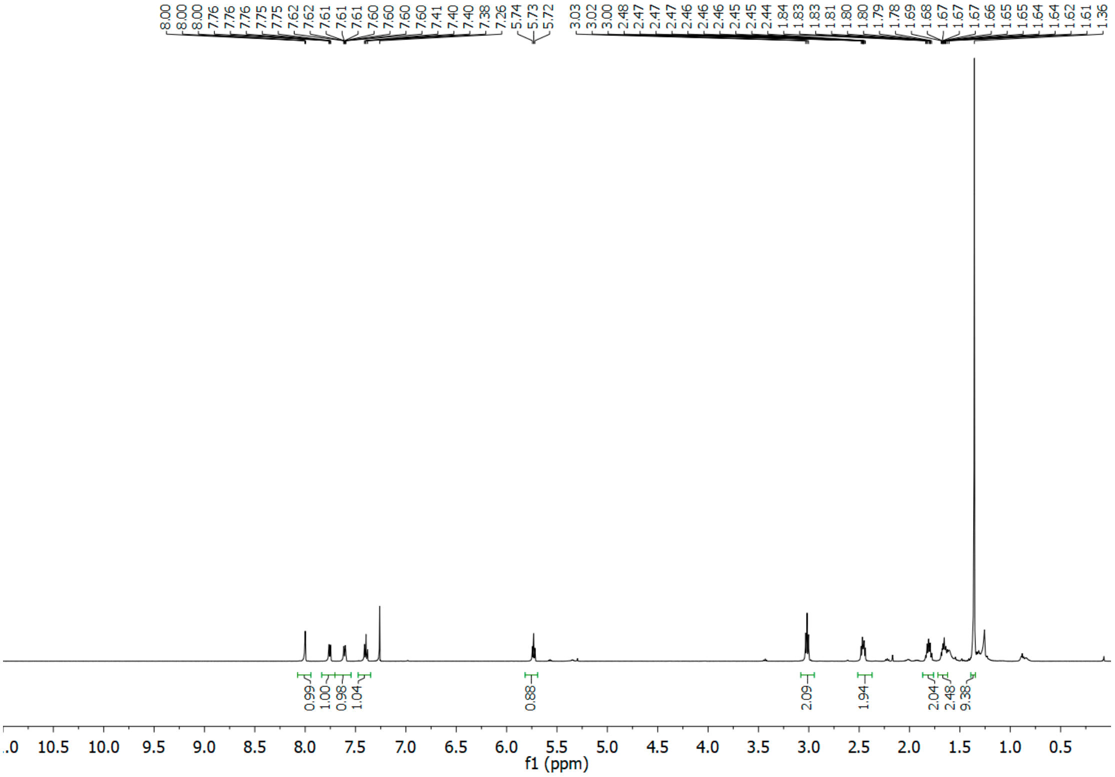

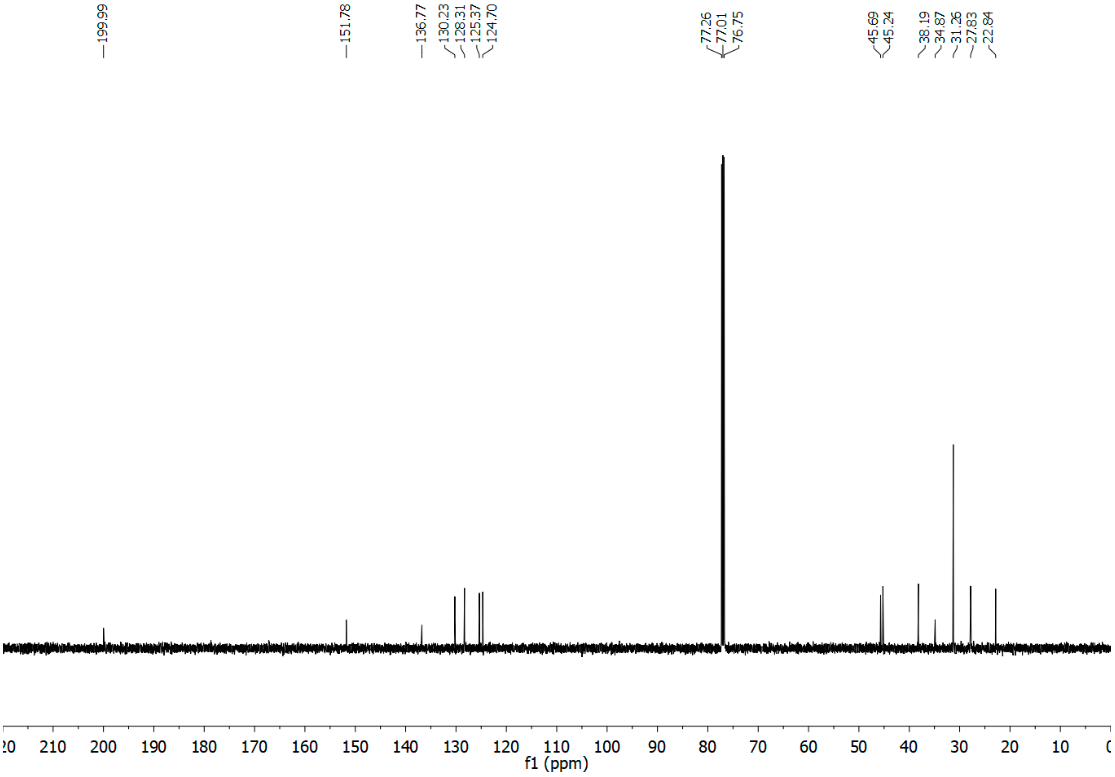

*Figure S12. ^{1}H NMR spectrum ($CDCl_3$, 400 MHz) and ^{13}C NMR spectrum ($CDCl_3$, 101 MHz) of **2g***

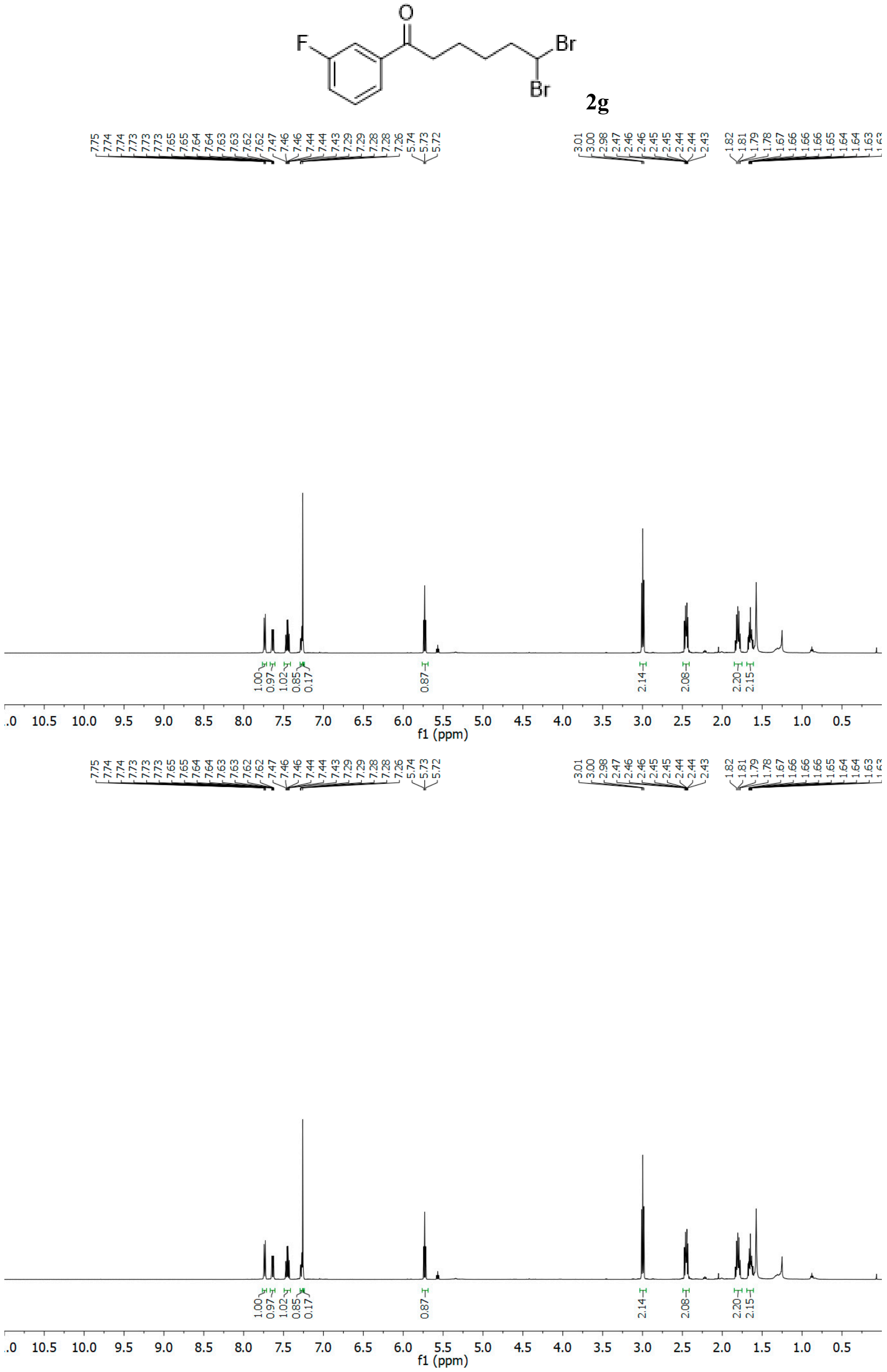

*Figure S13. ^{1}H NMR spectrum ($CDCl_3$, 400 MHz) and ^{13}C NMR spectrum ($CDCl_3$, 101 MHz) of **2h***

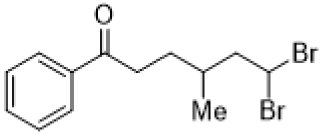

2h

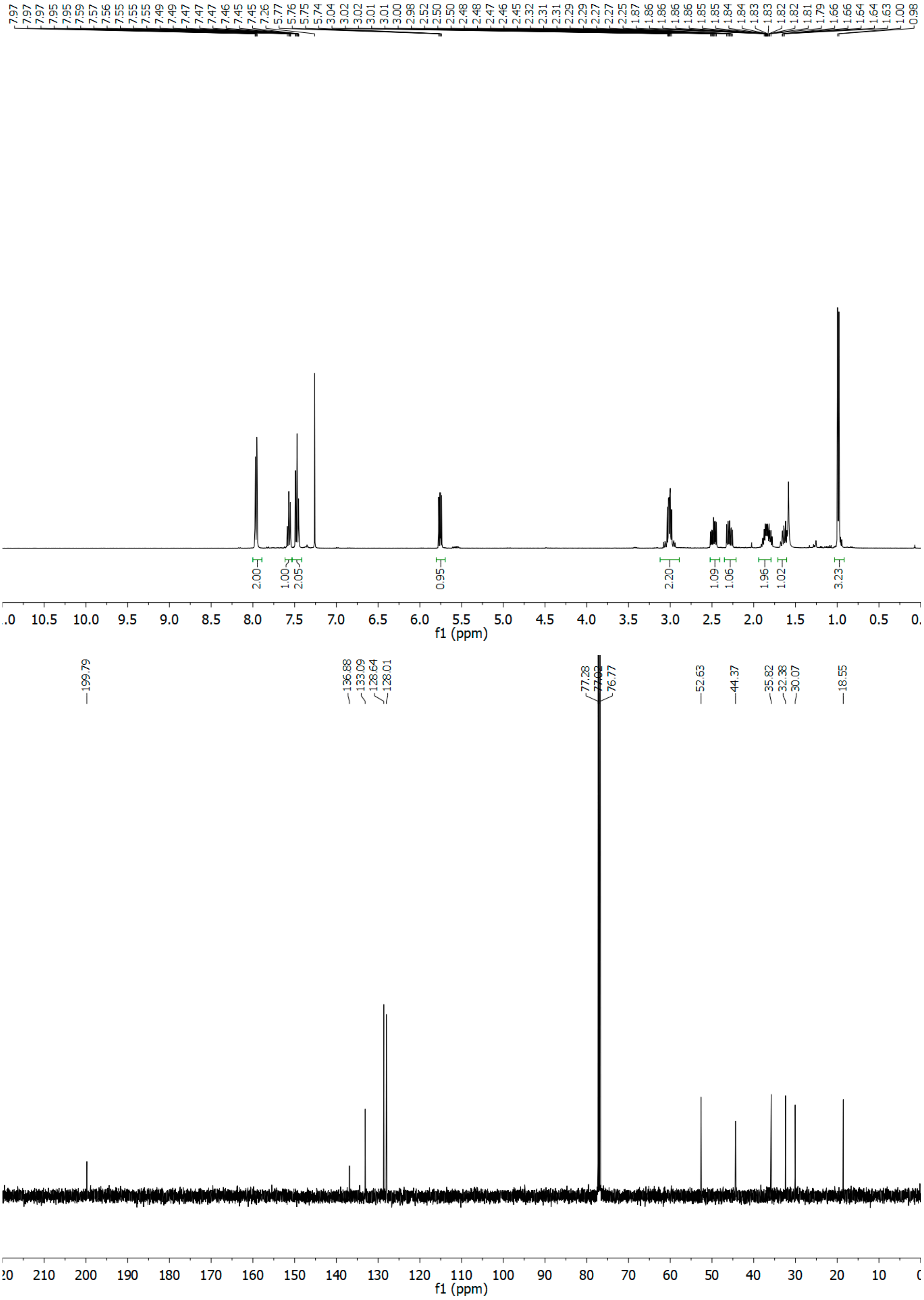

*Figure S14. ^{1}H NMR spectrum ($CDCl_3$, 400 MHz) and ^{13}C NMR spectrum ($CDCl_3$, 101 MHz) of **2i***

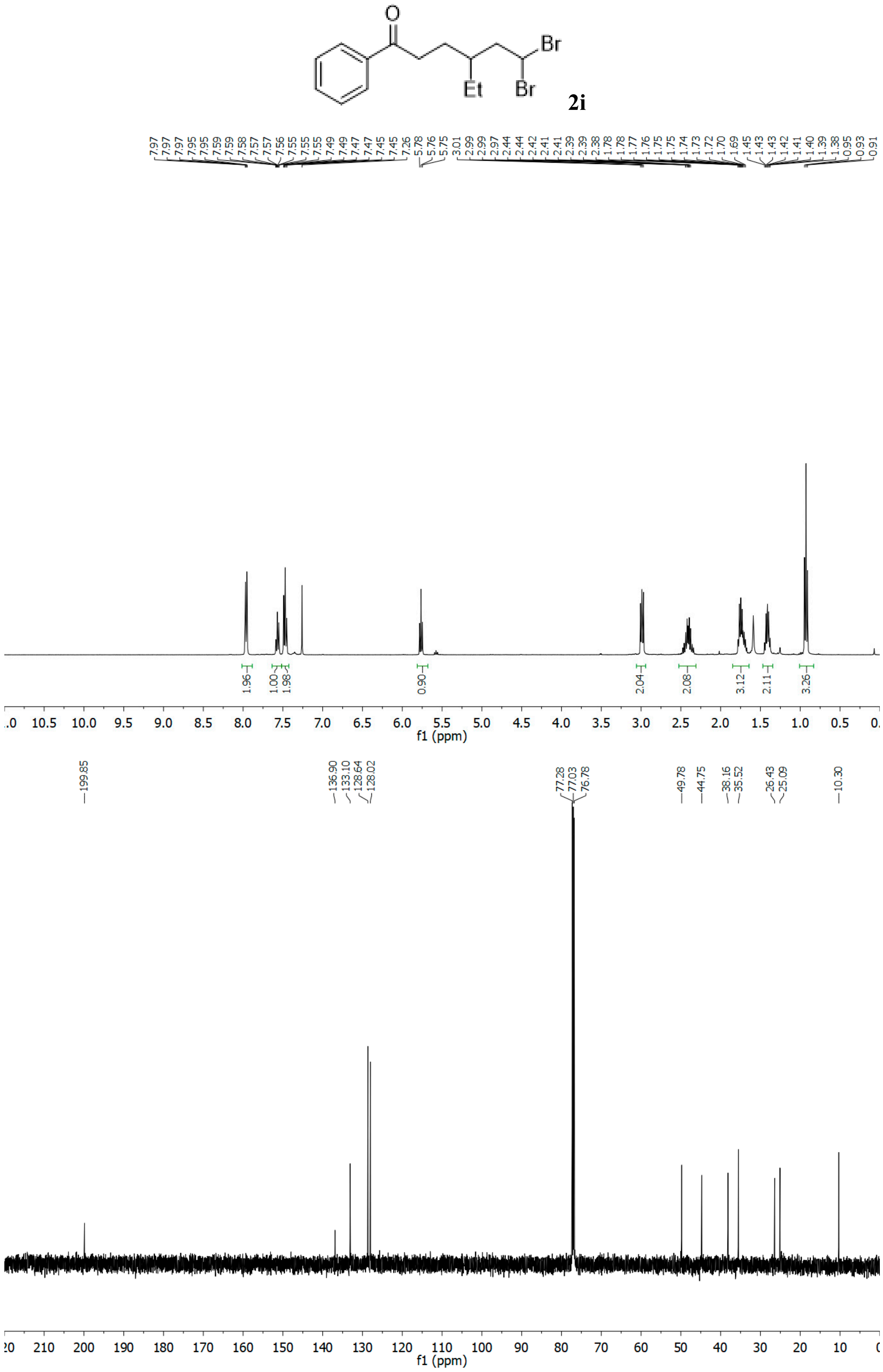

Figure S15. *^{1}H NMR spectrum (CDCl$_3$, 400 MHz) and ^{13}C NMR spectrum (CDCl$_3$, 101 MHz) of* ***2j***

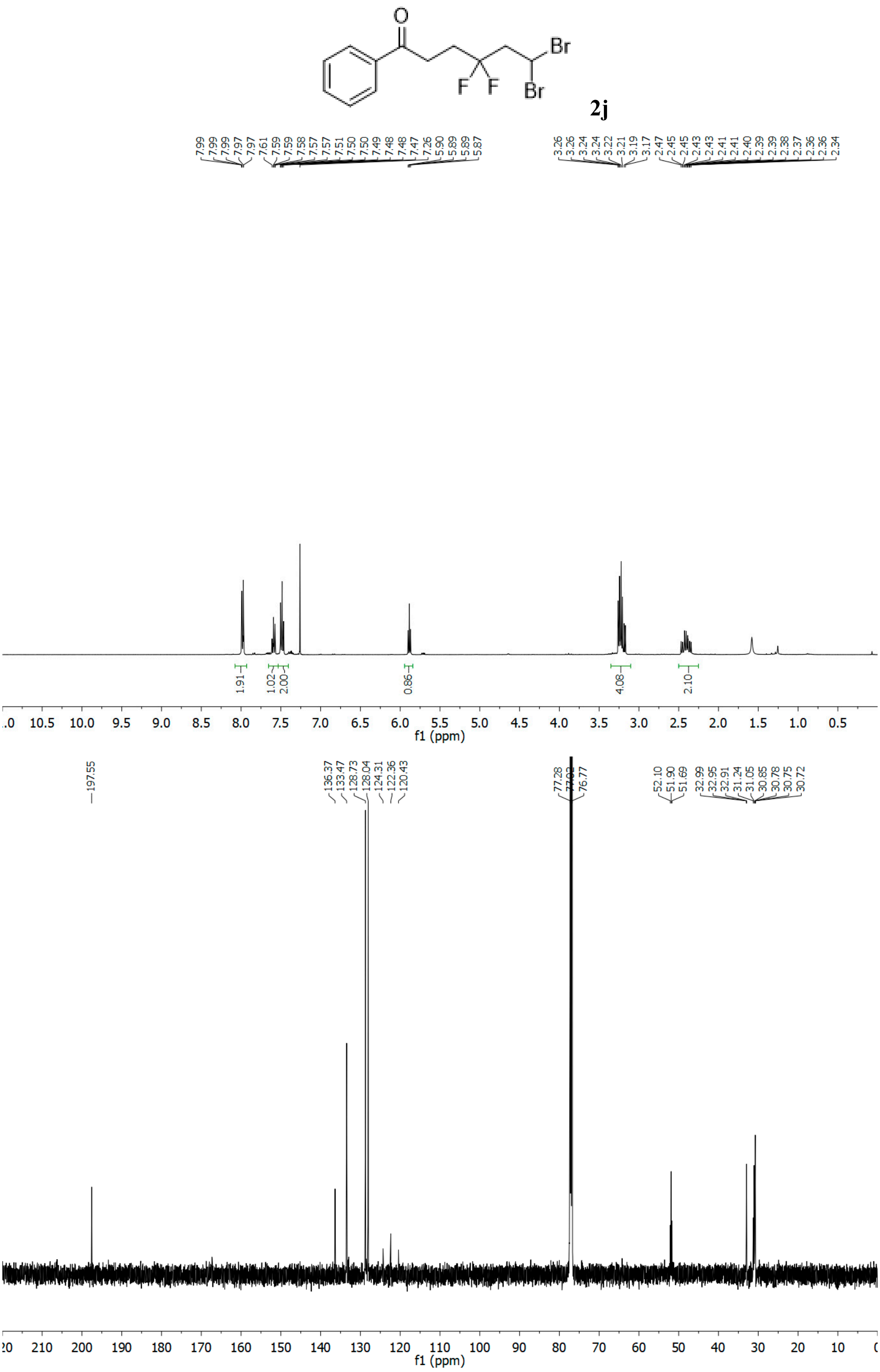

*Figure S16. ^{1}H NMR spectrum (CDCl$_3$, 400 MHz) and ^{13}C NMR spectrum (CDCl$_3$, 101 MHz) of **2k***

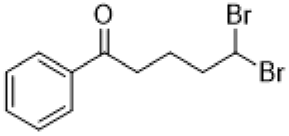

7a

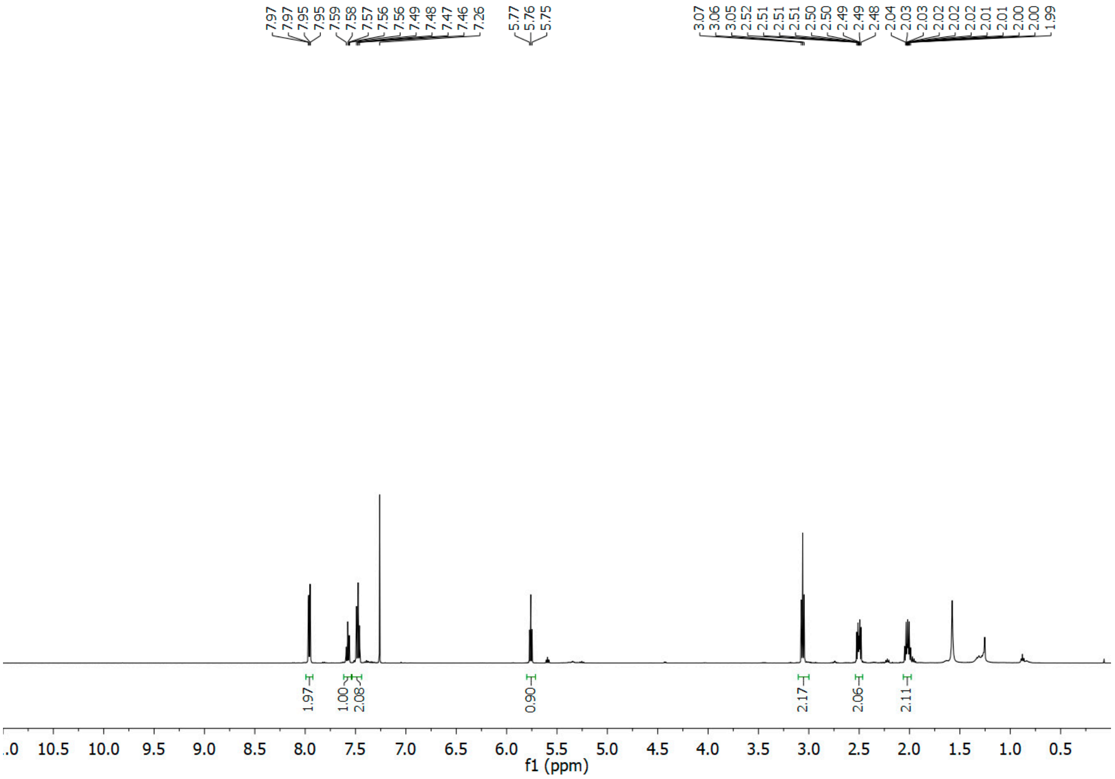

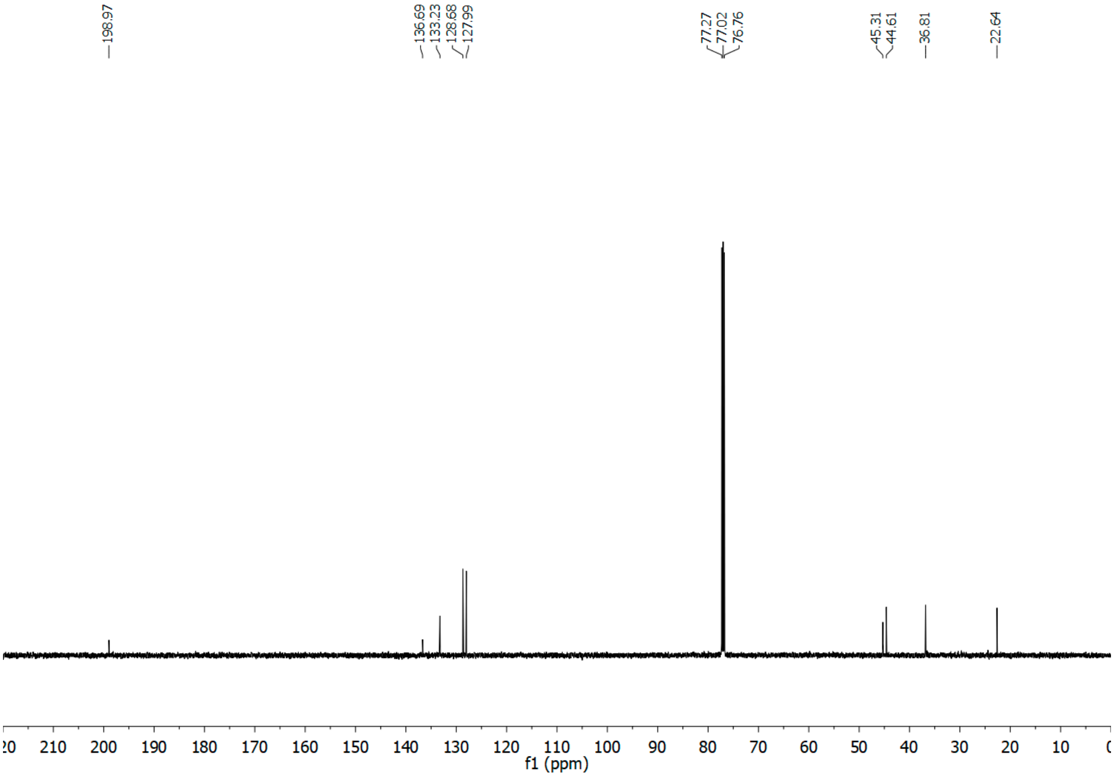

Figure S17. [1]*H NMR spectrum (CDCl*$_3$*, 400 MHz) and* [13]*C NMR spectrum (CDCl*$_3$*, 101 MHz) of* ***7a***

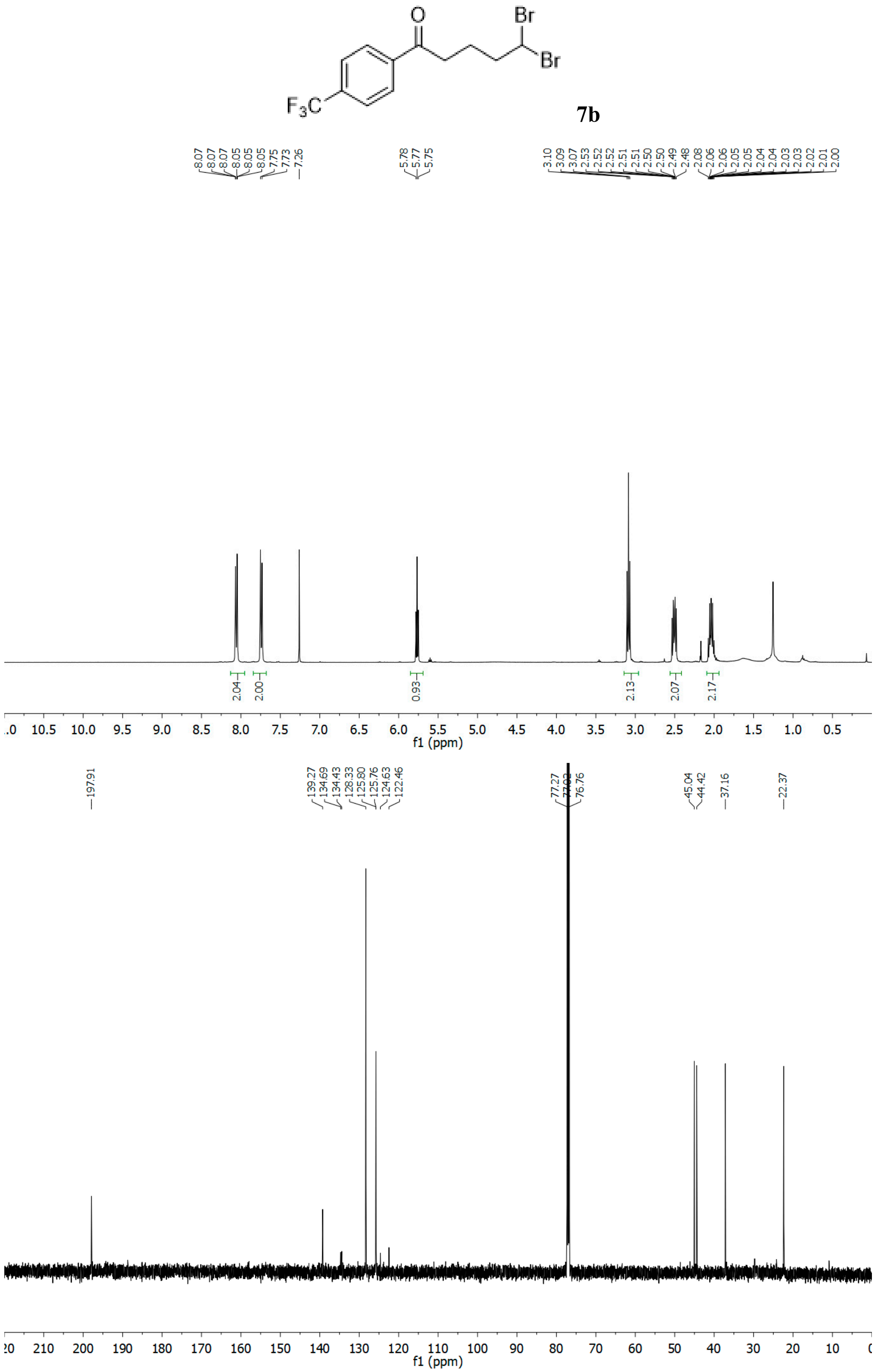

Figure S18. ^{1}H NMR spectrum ($CDCl_3$, 400 MHz) and ^{13}C NMR spectrum ($CDCl_3$, 101 MHz) of **7b**

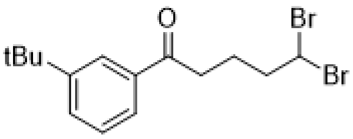

7c

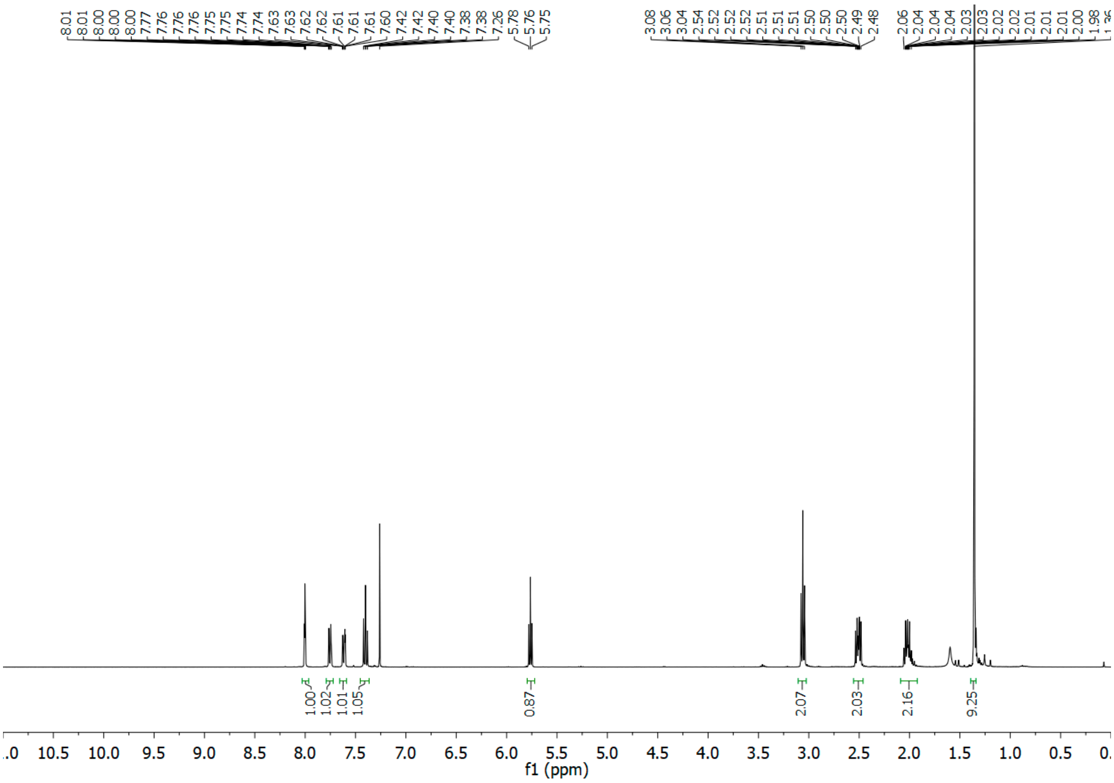

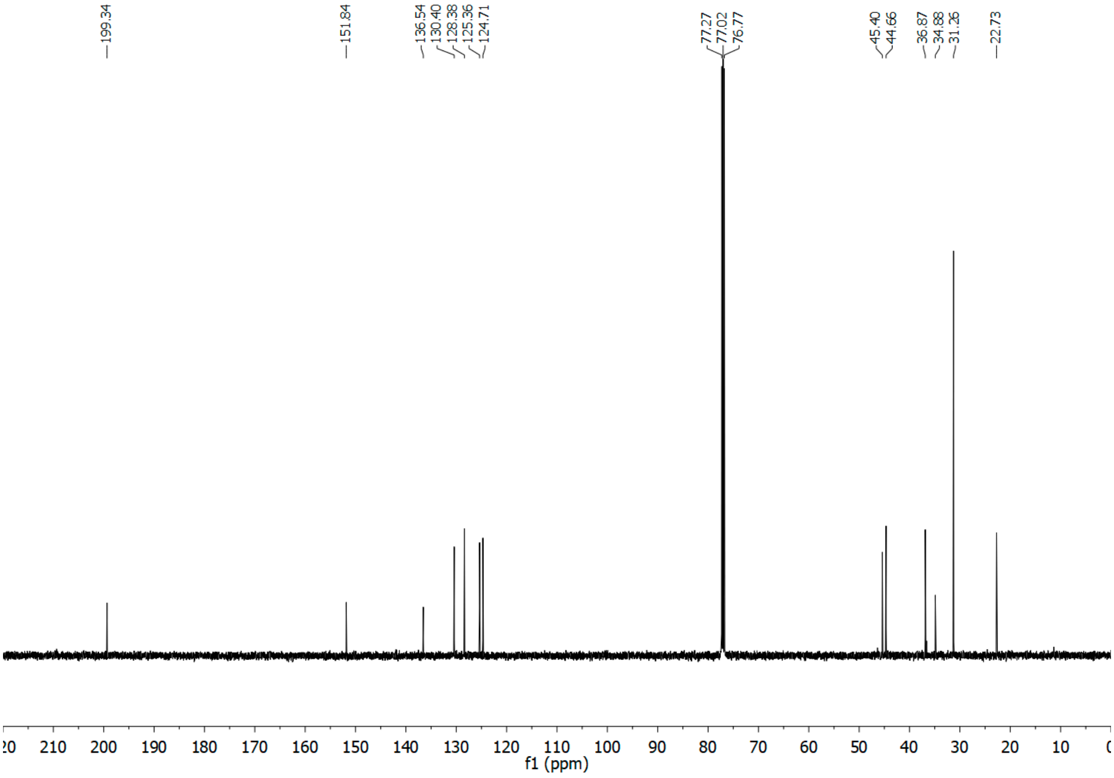

*Figure S19. ^{1}H NMR spectrum ($CDCl_3$, 400 MHz) and ^{13}C NMR spectrum ($CDCl_3$, 101 MHz) of **7c***

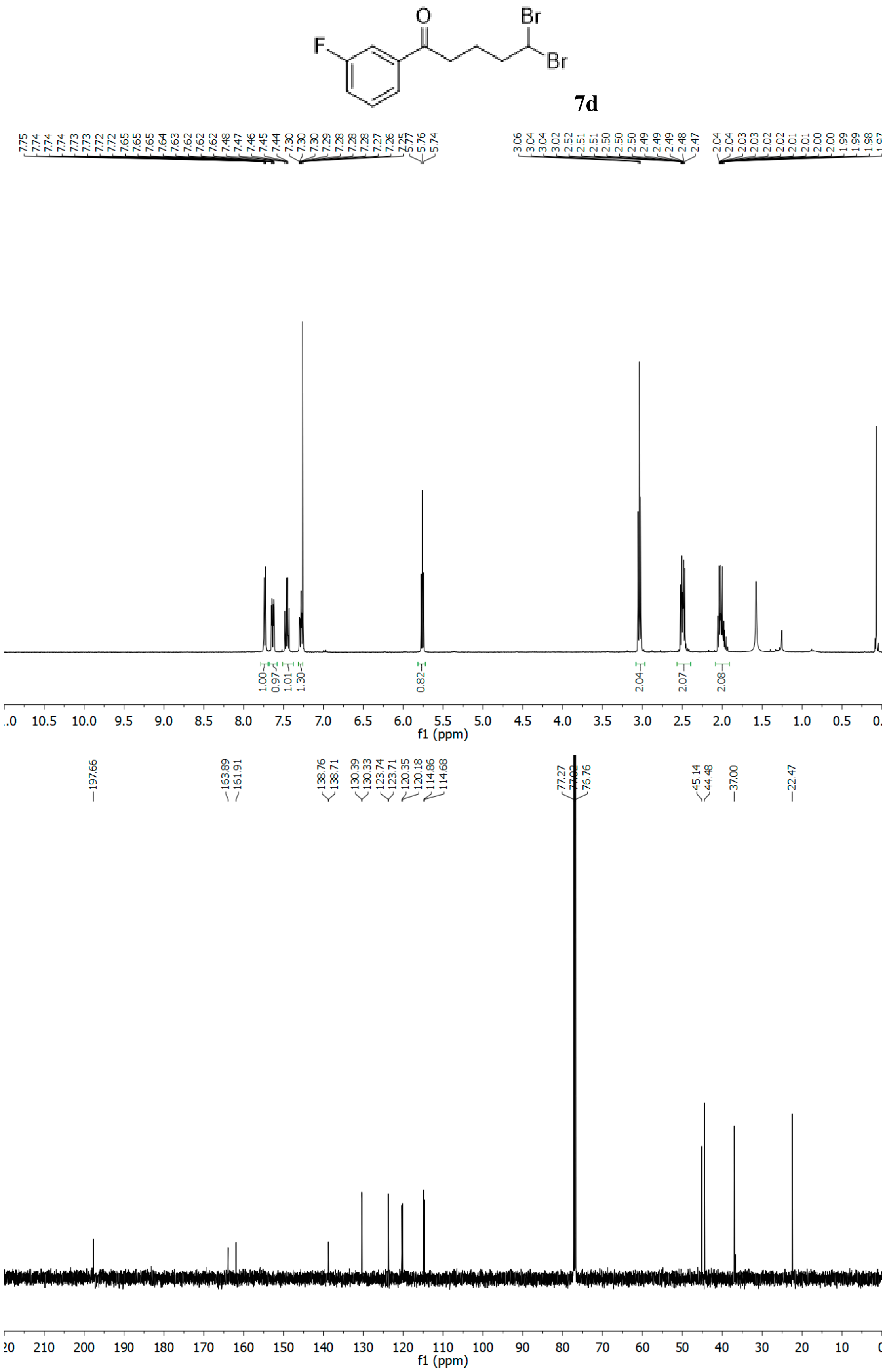

*Figure S20. ^{1}H NMR spectrum ($CDCl_3$, 400 MHz) and ^{13}C NMR spectrum ($CDCl_3$, 101 MHz) of **7d***

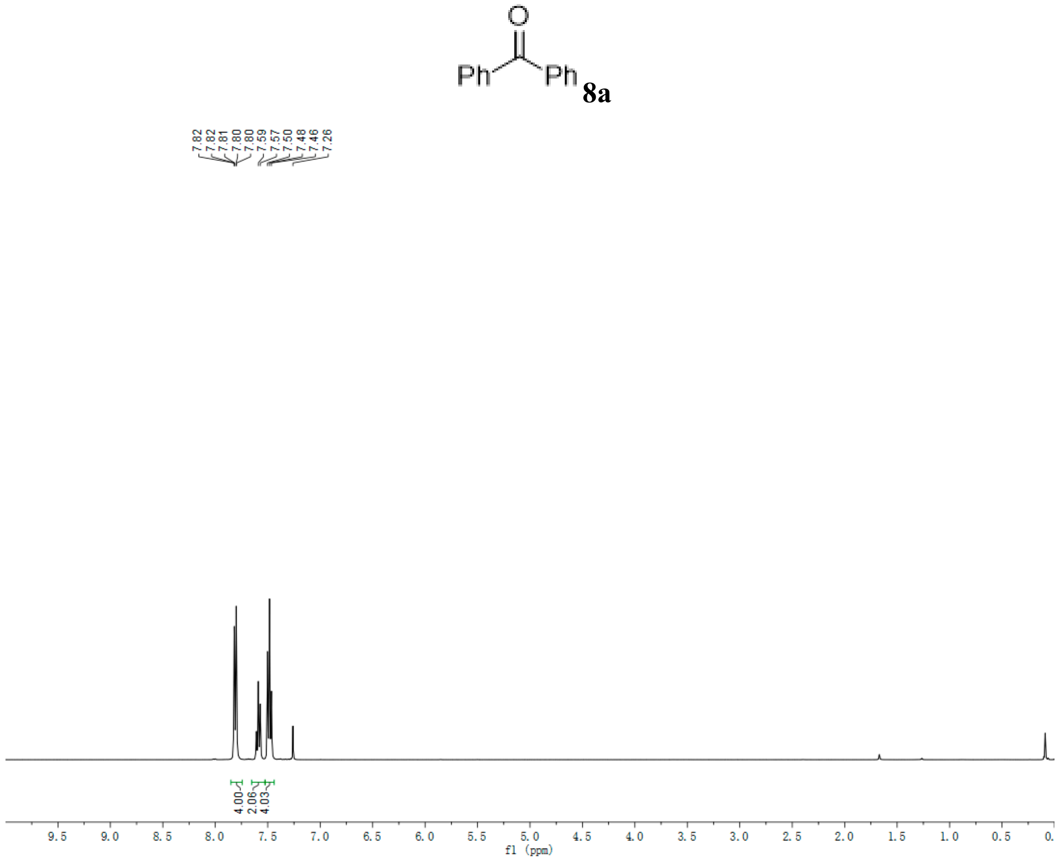

Figure S21. ^{1}H NMR spectrum ($CDCl_3$, 400 MHz) of ***8a***

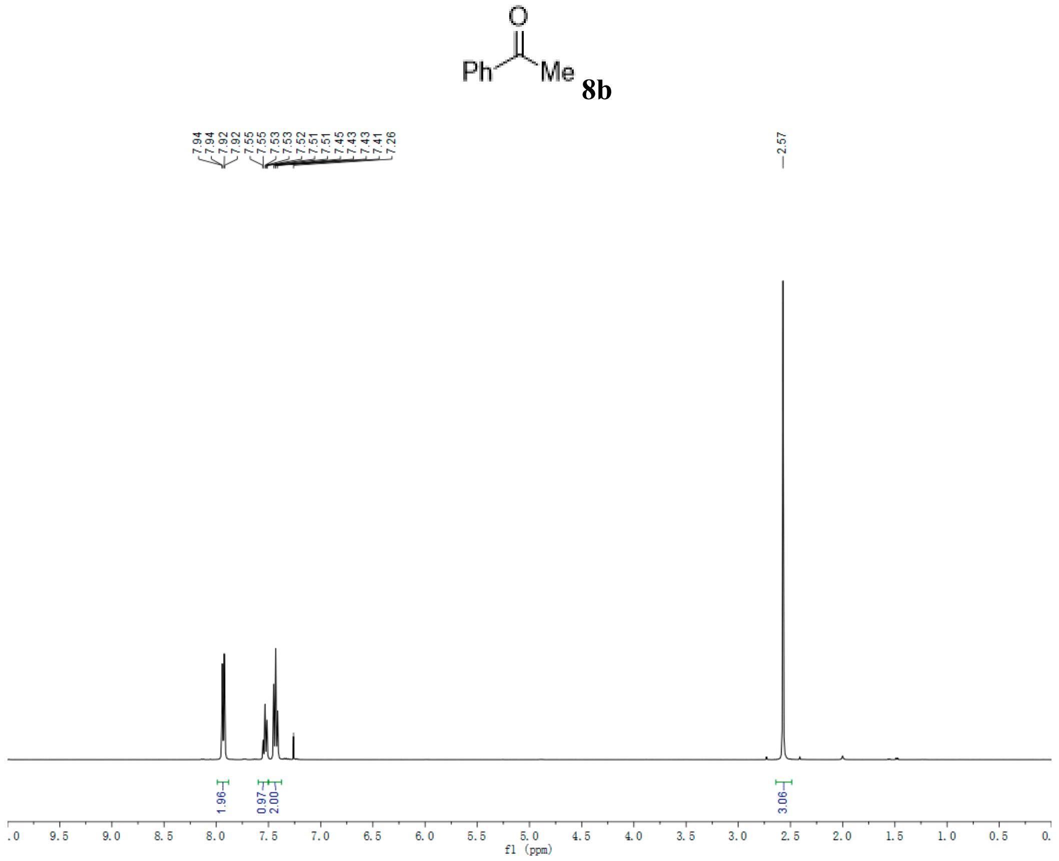

Figure S22. ^{1}H NMR spectrum ($CDCl_3$, 400 MHz) of ***8b***

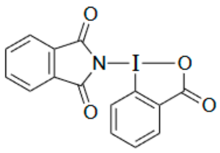

6

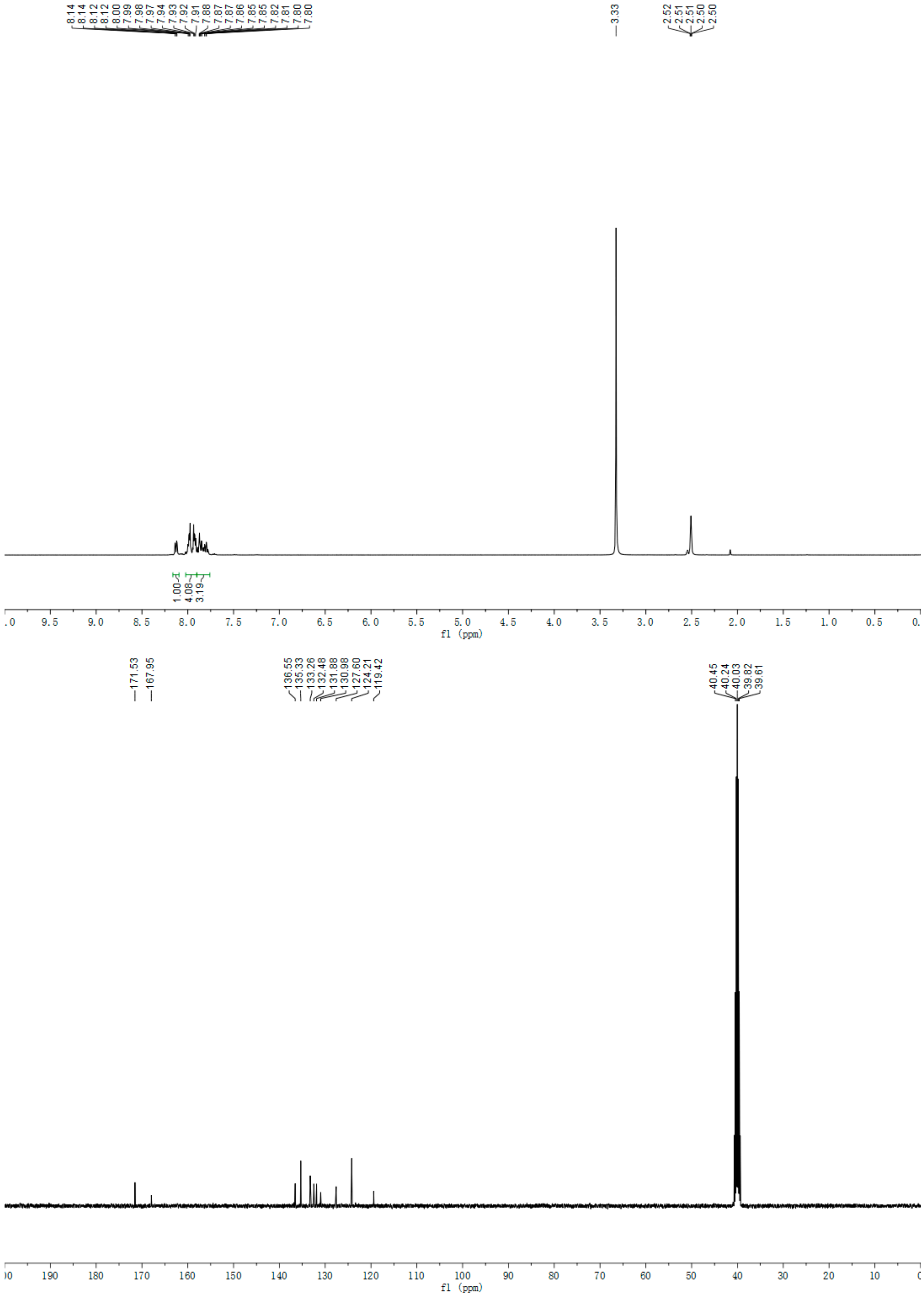

Figure S23. ^{1}H NMR spectrum ($CDCl_3$, 400 MHz) and ^{13}C NMR spectrum ($CDCl_3$, 101 MHz) of ***6***
